# Supplementary material for: Failure-to-rescue as a determinant of overall survival following resection for perihilar cholangiocarcinoma
Source: JHEP Rep. 2025 Oct 3;7(12):101615. doi: 10.1016/j.jhepr.2025.101615 (PMC12666550; doi:10.1016/j.jhepr.2025.101615)
Supplement: Multimedia component 1 [file mmc1.pdf]

Failure-to-rescue as a determinant of overall survival following resection for perihilar cholangiocarcinoma

Yawen Dong, Zhihao Li, Vanja Podrascanin, John E. Eaton, Sumera I. Ilyas, Gregory J. Gores, Susanne G. Warner, David M. Nagorney, Rory L. Smoot, Patrick P. Starlinger

Table of contents

Table S1……2

Table S2……3

Table S3……4

Fig. S1……7

Fig. S2……8

Table S4……9

Table S5……12

Fig. S3……13

Cause of Death in Failure to Rescue (FTR) Patients (N=15)	Total (N=15)	2000-2010 (N=7)	2011-2017 (N=5)	2017-2024 (N=3)
Primary posthepatectomy liver failure (PHLF)	2 (13.3%)	1 (14.3%)	0 (0.0%)	1 (33.3%)
Secondary PHLF, due to:<br>- Sepsis (n=3)<br>- Portal vein thrombosis (n=1)	4 (26.7%)	1 (14.3%)	2 (40.0%)	1 (33.3%)
Non-PHLF-related causes:<br>- Sepsis (n=4)<br>- Hemorrhage (n=2)<br>- Thromboembolic events (n=3)	9 (60.0%)	5 (71.4%)	3 (60.0%)	1 (33.3%)

Table S1. Summary of causes of death in patients with failure to rescue (FTR).

Rescue Interventions	2000-2010 (n=29)	2011-2017 (n=35)	2018-2024 (n=49)	Total (n=113)
ICU based rescue measures:	9 (31.0%)	11 (31.4%)	7 (14.3%)	27 (23.9%)
• Cardiorespiratory	3	4	3	10
• Bile leak/ PHLF	4	3	2	9
• Sepsis	1	1	1	3
• Bleeding	1	3	1	5
Relaparotomy	6 (20.7%)	11 (31.4%)	5 (10.2%)	22 (19.5%)
• Bile leak	3	4	3	10
• Intraabdominal hemorrhage	3	5	1	9
• Hepatic artery/ portal vein thrombosis	0	2	0	2
• Fascia dehiscence	0	0	1	1
Endoscopy based procedures:	0 (0.0%)	3 (8.6%)	2 (4.1%)	5 (4.4%)
• ERCP for bile leak	0	0	1	1
• EGD for GI bleeding	0	3	1	4
IR vascular procedures (e.g. coil embolization, PV stenting)	1 (3.5%)	1 (2.9%)	7 (14.3%)	9 (8.0%)
• Hepatic artery pseudoaneurysm	1	1	3	5
• Portal vein thrombosis	0	0	2	2
• Bleeding from right inferior phrenic artery	0	0	1	1
• Bleeding from gastroduodenal artery	0	0	1	1
IR PTCD placement for bile leak	3 (10.3%)	2 (5.7%)	15 (30.6%)	20 (17.7%)
Image guided drainage (e.g. CT/ US guided, paracentesis, thoracocentesis)	10 (34.5%)	7 (20.0%)	13 (26.5%)	30 (26.5%)
• Bilioma	2	1	4	7
• Abscess	5	5	7	17
• Pleural effusion	2	0	0	2
• Ascites	1	0	1	2
• Infected hematoma	0	1	1	2

Table S2. Rescue interventions following major complications in resected pCCA patients by era (2000-2010 vs 2011-2017 vs 2018-2024). Percentages are calculated per time period. PHLF = Post-hepatectomy liver failure, ERCP = Endoscopic retrograde cholangiopancreatography, EGD = Esophagogastroduodenoscopy, GI = Gastrointestinal, IR = Interventional radiology, PV = Portal vein.

Variable	Major Complications (N=81)	Rescued group (N=66)	Failure to rescue group (N=15)	p value
Sex				0.331
Male	52 (64.2%)	44 (66.7%)	8 (53.3%)	
Female	29 (35.8%)	22 (33.3%)	7 (46.7%)	
Age (years)	68.0 (56.5-73.0)	68.0 (57.0-73.0)	69.0 (57.0-75.5)	0.212
BMI (kg/m^2)	25.6 (23.2-30.0)	25.1 (23.0-28.7)	30.0 (25.8-39.4)	0.002
ECOG				0.250
• 0	58 (71.6%)	49 (74.2%)	9 (60.0%)	
• > 0	23 (28.4%)	17 (25.8%)	6 (40.0%)	
Bilirubin (mg/dl)	0.9 (0.5-2.6)	0.9 (0.5-2.6)	1.1 (0.6-2.6)	0.209
APRI+ALBI	-2.17 (-2.49 - -1.55)	-2.24 (-2.54 - -1.52)	-2.20 (-2.42 - -0.73)	0.023
Prothrombin time (sec)	11.5 (10.5-12.78)	11.6 (10.9-12.8)	11.3 (10.2-12.8)	0.804
Platelets (x10^9/L)	251.0 (200.0-322.0)	242.0 (197.0-312.0)	243.0 (180.5-379.0)	0.597
INR	1.0 (1.0-1.1)	1.1 (1.0-1.2)	1.0 (1.0-1.2)	0.902
AST (U/L)	54.0 (34.5-83.5)	59.0 (35.0-88.0)	57.0 (43.0-112.0)	0.316
ALT (U/L)	61.0 (37.8-104.5)	61.0 (37.0-110.0)	67.0 (48.0-105.0)	0.681
Albumin (g/dl)	4.0 (3.7-4.2)	4.0 (3.7-4.3)	3.9 (3.6-4.1)	0.038
CA19-9 (U/ml)	108.5 (36.0-372.8)	107.0 (38.0-374.0)	239.0 (36.0-713.0)	0.635
Bismuth-Corlette				0.063
• I	2 (2.5%)	1 (1.5%)	1 (6.7%)	
• II	5 (6.2%)	5 (7.6%)	0 (0.0%)	
• IIIa	40 (49.4%)	35 (53.0%)	5 (33.3%)	
• IIIb	27 (33.3%)	18 (27.3%)	9 (60.0%)	
• IV	7 (8.6%)	7 (10.6%)	0 (0.0%)	
PreOP stenting	70 (86.4%)	55 (83.3%)	15 (100%)	0.089
Portal vein embolization	16 (19.8%)	15 (22.7%)	1 (6.7%)	0.158
Time period of surgery				0.181
• 2000-2010	24 (29.6%)	17 (25.8%)	7 (46.7%)	
• 2011-2017	26 (32.1%)	21 (31.8%)	5 (33.3%)	
• 2018-2024	31 (38.3%)	28 (42.4%)	3 (20.0%)	

Type of liver resection				0.033
• Right hepatectomy	26 (32.1%)	22 (33.3%)	4 (26.7%)	
• Extended right hepatectomy	24 (29.6%)	21 (31.8%)	3 (20.0%)	
• Left hepatectomy	19 (23.5%)	11 (16.7%)	8 (53.3%)	
• Extended left hepatectomy	9 (11.1%)	9 (13.6%)	0 (0.0%)	
• Central hepatectomy	3 (3.7%)	3 (4.6%)	0 (0.0%)	
Vascular reconstruction	19 (23.5%)	16 (24.2%)	3 (20.0%)	0.726
TNM Stage (AJCC 8th edition)				0.671
• Stage 0	1 (1.2%)	1 (1.5%)	0 (0.0%)	
• Stage 1	10 (12.3%)	9 (13.6%)	1 (6.7%)	
• Stage 2	29 (35.8%)	25 (37.9%)	4 (26.7%)	
• Stage 3	35 (43.2%)	26 (39.4%)	9 (60.0%)	
• Stage 4	6 (7.4%)	5 (7.6%)	1 (6.7%)	
Tumor size (cm)	2.9 (2.3-4.0)	2.8 (2.2-3.4)	3.5 (2.5-5.0)	0.513
Lymph node (LN) status				0.454
• N0	39 (48.1%)	33 (50.0%)	6 (40.0%)	
• N1	36 (44.4%)	27 (40.9%)	9 (60.0%)	
• N2	2 (2.5%)	2 (3.0%)	0 (0.0%)	
• Nx	4 (4.9%)	4 (6.1%)	0 (0.0%)	
Histologic grade				0.868
• G1	12 (14.8%)	9 (13.6%)	0 (0.0%)	
• G2	30 (37.0%)	26 (39.4%)	3 (20.0%)	
• G3	33 (40.7%)	26 (39.4%)	7 (46.7%)	
• G4	5 (6.2%)	4 (6.1%)	1 (6.7%)	
• NA	1 (1.2%)	1 (1.5%)	0 (0.0%)	
Resection margin				0.489
• R0	65 (80.2%)	52 (78.8%)	13 (86.7%)	
• R1	16 (19.8%)	14 (21.2%)	2 (13.3%)	
Estimated blood loss (ml)	700.0 (500.0-1400.0)	700.0 (500.0-1500.0)	800.0 (350.0-1150.0)	0.981
PostOP stay (days)	12.0 (9.0-19.0)	12.0 (9.0-19.0)	7.0 (6.0-16.0)	0.214

Neoadjuvant therapy	9 (11.1%)	8 (12.1%)	1 (6.7%)	0.544
PHLF Grade B/ C	34 (42.0%)	27 (40.9%)	7 (46.7%)	0.530

Table S3. Comparison of major complications, 90-day mortality, and failure to rescue (FTR) rates between rescued and failure to rescue (FTR) patients following major hepatectomy for pCCA.

Continuous variables are presented as medians with interquartile ranges (IQRs) and were compared using the Mann-Whitney U test for pairwise comparisons. Categorical variables are reported as counts and percentages and were compared using the Chi-square test or Fisher’s exact test, as appropriate. A p-value <0.05 was considered statistically significant; significant p-values are indicated in bold.

BMI body mass index, ECOG Eastern Cooperative Oncology Group, APRI Aspartate Aminotransferase to Platelet Ratio Index, ALBI Albumin-Bilirubin Score, INR international normalized ratio, AST Aspartate Aminotransferase, ALT Alanine Aminotransferase, CA7❺_❺Carbohydrate Antigen 19-9, PVE portal vein embolization, pCCA.perihilar cholangiocarcinoma.

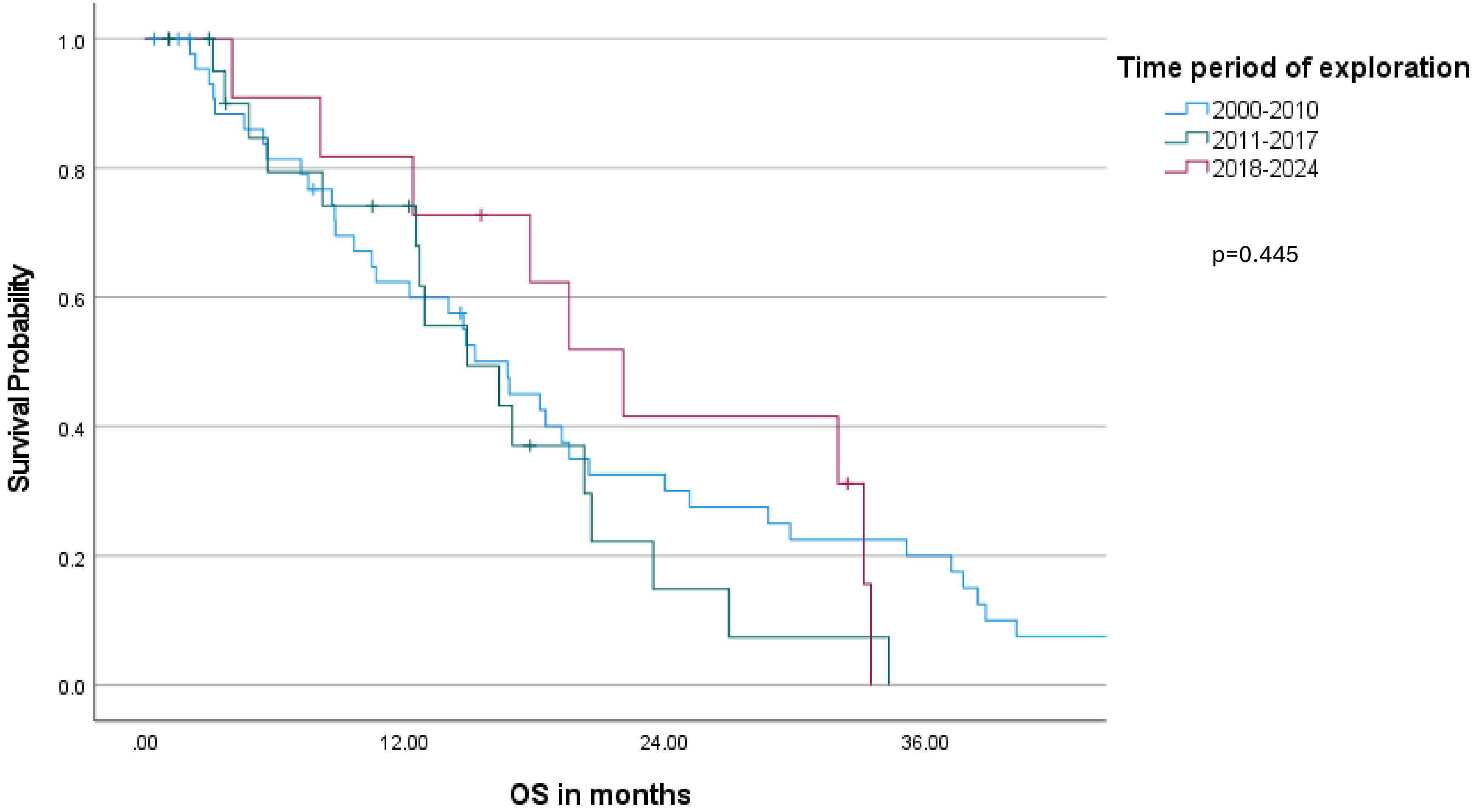

Number at risk

2000-2010	46	26	12	8
2011-2017	24	13	2	0
2018-2024	11	9	4	0

Fig. S1. Kaplan Meier Curve demonstrating OS of all pCCA dropout patients stratified by time period of surgical exploration.

Statistical analysis was performed using Kaplan-Meier survival estimates and log-rank test. A p-value <0.05 was considered statistically significant, and significant p-values are indicated in bold, while non-significant p-values are shown without formatting.

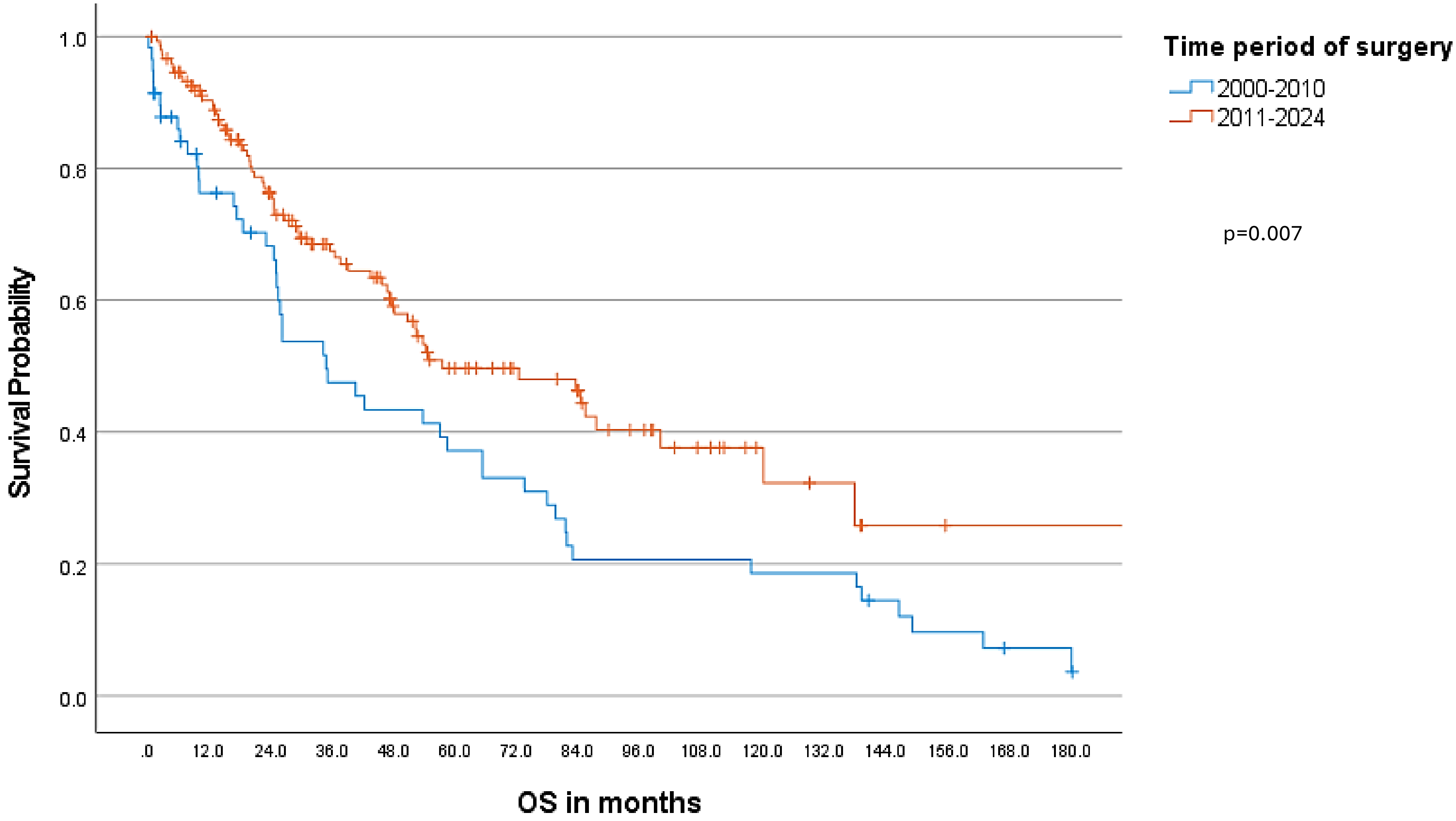

Number at risk

2000-2010	58	39	33	23	21	18	16	10	10	10	9	9	6	4	2	1
2011-2024	149	123	91	67	52	38	30	25	18	12	7	5	2	1	1	1

Fig. S2. Assessment of the impact of adjuvant therapy on OS in resected pCCA patients. Comparison between a historical cohort (2000–2010) and a recent cohort (2011–2024), using 2010 as the cutoff year to reflect the increasing adoption of adjuvant therapy thereafter. Statistical analysis was performed using Kaplan-Meier survival estimates and log-rank test. A p-value <0.05 was considered statistically significant, and significant p-values are indicated in bold, while non-significant p-values are shown without formatting.

Variable	No adjuvant therapy (N=111)	Adjuvant therapy (N=96)	p value
Sex			0.797
Male	69 (62.2%)	58 (60.4%)	
Female	42 (37.8%)	38 (39.6%)	
Age (years)	68.0 (60.0-73.5)	63.0 (52.0-71.0)	0.001
BMI (kg/m^2)	25.6 (22.6-28.9)	25.2 (22.8-29.7)	0.870
ECOG			0.148
0	76 (68.5%)	77 (80.2%)	
> 0	35 (31.5%)	19 (19.8%)	
Bilirubin (mg/dl)	1.1 (0.6-3.2)	0.9 (0.5-2.1)	0.132
APRI+ALBI	-1.85 (-2.42 - -1.35)	-2.23 (-2.62 - -1.65)	0.037
Prothrombin time (sec)	11.6 (10.8-12.7)	11.7 (11.2-12.2)	0.099
Platelets (x10^9/L)	243.0 (197.5-307.0)	256.0 (197.0-307.0)	0.919
INR	1.0 (1.0-1.2)	1.0 (1.0-1.1)	0.204
AST (U/L)	62.0 (45.0-87.5)	51.0 (34.0-84.0)	0.103
ALT (U/L)	69.0 (48.5-111.5)	65.0 (44.0-119.0)	0.794
Albumin (g/dl)	3.9 (3.6-4.2)	4.1 (3.8-4.3)	0.046
CA19-9 (U/ml)	145.0 (36.0-514.0)	73.0 (22.0-259.0)	0.181
Bismuth-Corlette			0.010
I	2 (1.8%)	3 (3.1%)	
II	3 (2.7%)	8 (8.3%)	
IIIa	57 (51.4%)	31 (32.3%)	
IIIb	45 (40.5%)	42 (43.8%)	
IV	4 (3.6%)	12 (12.5%)	
PreOP stenting	103 (92.8%)	83 (86.5%)	0.132
Portal vein embolization	13 (11.7%)	11 (11.5%)	0.955
Time period of surgery			<0.001
2000-2010	45 (40.6%)	13 (13.5%)	
2011-2017	42 (37.8%)	31 (32.3%)	
2018-2024	24 (21.6%)	52 (54.2%)	
Type of liver resection			0.068
Right hepatectomy	43 (38.7%)	22 (22.9%)	
Extended right hepatectomy	18 (16.3%)	20 (20.8%)	

Left hepatectomy<br>Extended left hepatectomy<br>Central hepatectomy	40 (36.0%)<br>8 (7.2%)<br>2 (1.8%)	36 (37.5%)<br>16 (16.7%)<br>2 (2.1%)	
Vascular reconstruction	17 (15.3%)	20 (20.8%)	0.301
TNM Stage (AJCC 8th edition)<br>Stage 0<br>Stage 1<br>Stage 2<br>Stage 3<br>Stage 4	 1 (0.9%)<br>16 (14.4%)<br>42 (37.9%)<br>49 (44.1%)<br>3 (2.7%)	 0 (0.0%)<br>10 (10.4%)<br>26 (27.0%)<br>54 (56.3%)<br>6 (6.3%)	0.020
Tumor size (cm)	2.7 (2.2-3.5)	2.9 (2.3-3.6)	0.071
Lymph node (LN) status<br>N0<br>N1<br>N2<br>Nx	 71 (64.0%)<br>39 (35.1%)<br>1 (0.9%)<br>0 (0.0%)	 31 (32.3%)<br>55 (57.3%)<br>2 (2.1%)<br>8 (8.3%)	<0.001
Histologic grade<br>G1<br>G2<br>G3<br>G4<br>NA	 11 (9.9%)<br>46 (41.4%)<br>48 (43.2%)<br>5 (4.5%)<br>1 (0.9%)	 13 (13.6%)<br>51 (53.1%)<br>31 (32.3%)<br>1 (1.0%)<br>0 (0.0%)	0.040
Resection margin<br>R0<br>R1	 98 (88.3%)<br>13 (11.7%)	 68 (70.8%)<br>28 (29.2%)	0.002
Estimated blood loss (ml)	700.0 (425.0-1225.0)	600.0 (350.0-1200.0)	0.921
PostOP stay (days)	8.0 (5.0-15.0)	6.0 (4.0-10.0)	0.004
Neoadjuvant therapy	12 (10.8%)	11 (11.5%)	0.882
PHLF Grade B/ C	28 (25.2%)	9 (9.4%)	0.003
Major complications	55 (49.5%)	26 (27.1%)	<0.001
90d mortality	15 (13.5%)	0 (0.0%)	<0.001

Table S4. Baseline characteristics and clinicopathological parameters of pCCA patients stratified by receipt of adjuvant therapy. Continuous variables are presented as medians with interquartile ranges (IQRs) and were compared using the Mann-Whitney U test for pairwise comparisons. Categorical variables are reported as counts and percentages and were compared using the Chi-square test or Fisher's exact test, as appropriate. A p-value <0.05 was considered statistically significant; significant p-values are indicated in bold.

Time period	No Adjuvant Therapy Cohort (N=111)				Adjuvant Therapy Cohort (N=96)			
	Low risk & major complications (N=25)	Low risk w/o major complications (N=30)	High risk & major complications (N=30)	High risk w/o major complications (N=26)	Low risk & major complications (N=5)	Low risk w/o major complications (N=17)	High risk & major complications (N=21)	High risk w/o major complications (N=53)
2000-2010	9 (36.0%)	9 (30.0%)	13 (43.4(%)	14 (58.9%)	0 (0.0%)	0 (0.0%)	2 (9.5%)	11(20.8%)
2011-2017	9 (36.0%)	16 (53.3%)	10 (33.3%)	7 (26.9%)	2 (40.0%)	6 (35.3%)	5 (23.8%)	18 (34.0%)
2018-2024	7 (28.0%)	5 (16.7%)	7 (23.3%)	5 (19.2%)	3 (60.0%)	11 (64.7%)	14 (66.7%)	24 (45.2%)

Table S5. Distribution of patients in the adjuvant therapy cohort (N=96) and no adjuvant therapy cohort (N = 111), stratified by oncological risk profile (low vs. high risk) and presence of major postoperative complications across surgical time periods. Risk categorization was based on established adverse pathological features (e.g., nodal positivity, R1 resection, high-grade tumors), and major complications were defined as Clavien-Dindo grade ≥ III.

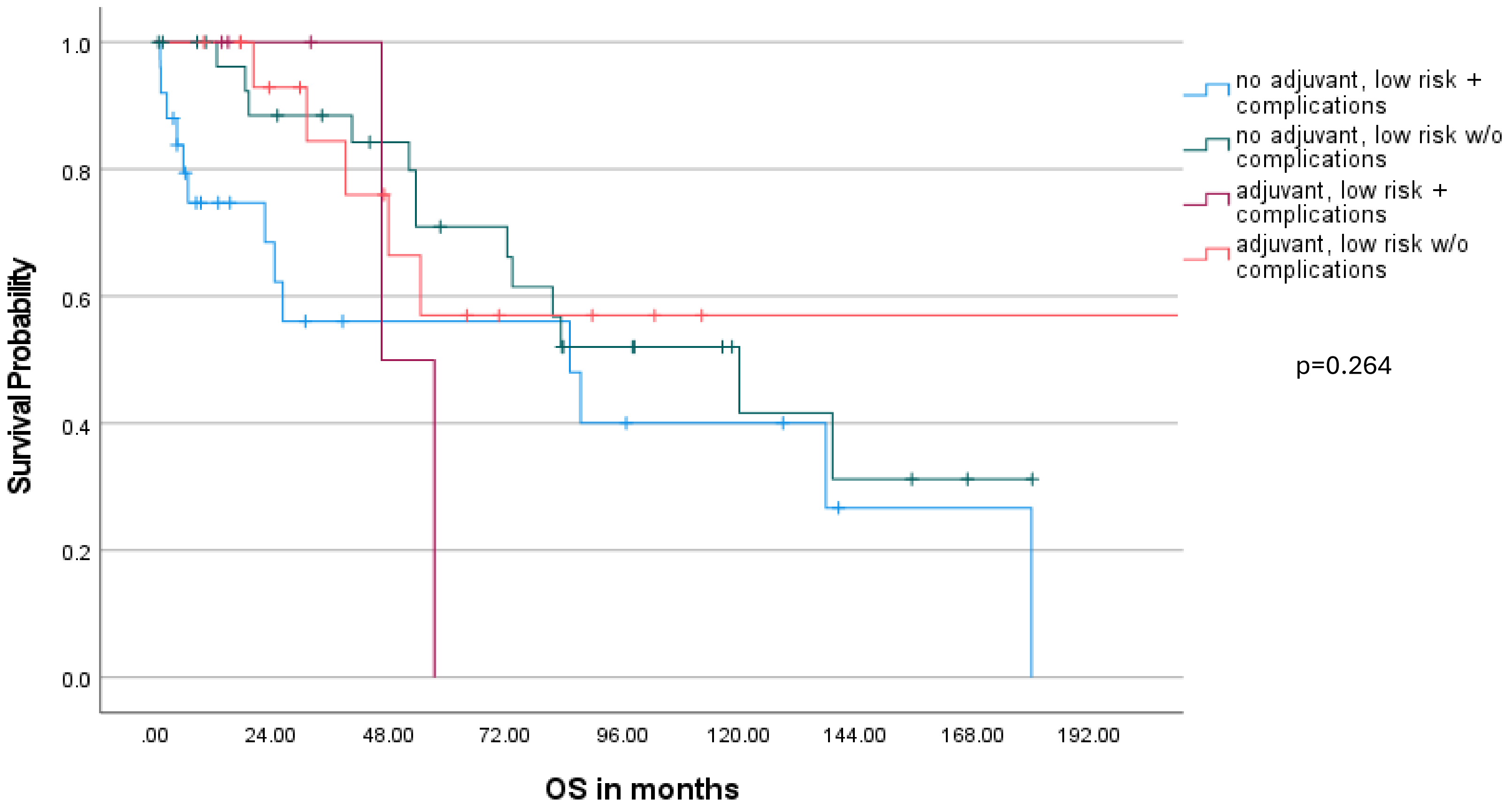

Number at risk

No adjuvant, low risk & complications	25	11	7	7	5	4	1	1	0
No adjuvant, low risk w/o complications	30	23	19	15	9	4	3	1	0
Adjuvant, low risk & complications	5	3	1	0	0	0	0	0	0
Adjuvant, low risk w/o complications	17	12	7	4	3	1	1	1	1

Fig. S3. Kaplan Meier Curve of OS in low risk profile patients stratified by receipt of adjuvant therapy and major complications. Statistical analysis was performed using Kaplan-Meier survival estimates and log-rank test. A p-value <0.05 was considered statistically significant, and significant p-values are indicated in bold, while non-significant p-values are shown without formatting.
