# Supplementary material for: Failure-to-rescue as a determinant of overall survival following resection for perihilar cholangiocarcinoma
Source: JHEP Rep. 2025 Oct 3;7(12):101615. doi: 10.1016/j.jhepr.2025.101615 (PMC12666550; doi:10.1016/j.jhepr.2025.101615)
Supplement: Multimedia component 4 [file mmc4.pdf]

Failure-to-rescue as a determinant of overall survival following resection for perihilar cholangiocarcinoma

Authors

Yawen Dong, Zhihao Li, Vanja Podrascanin, …, David M. Nagorney, Rory L. Smoot, Patrick P. Starlinger

Correspondence

starlinger.patrick@mayo.edu (P.P. Starlinger).

Graphical abstract

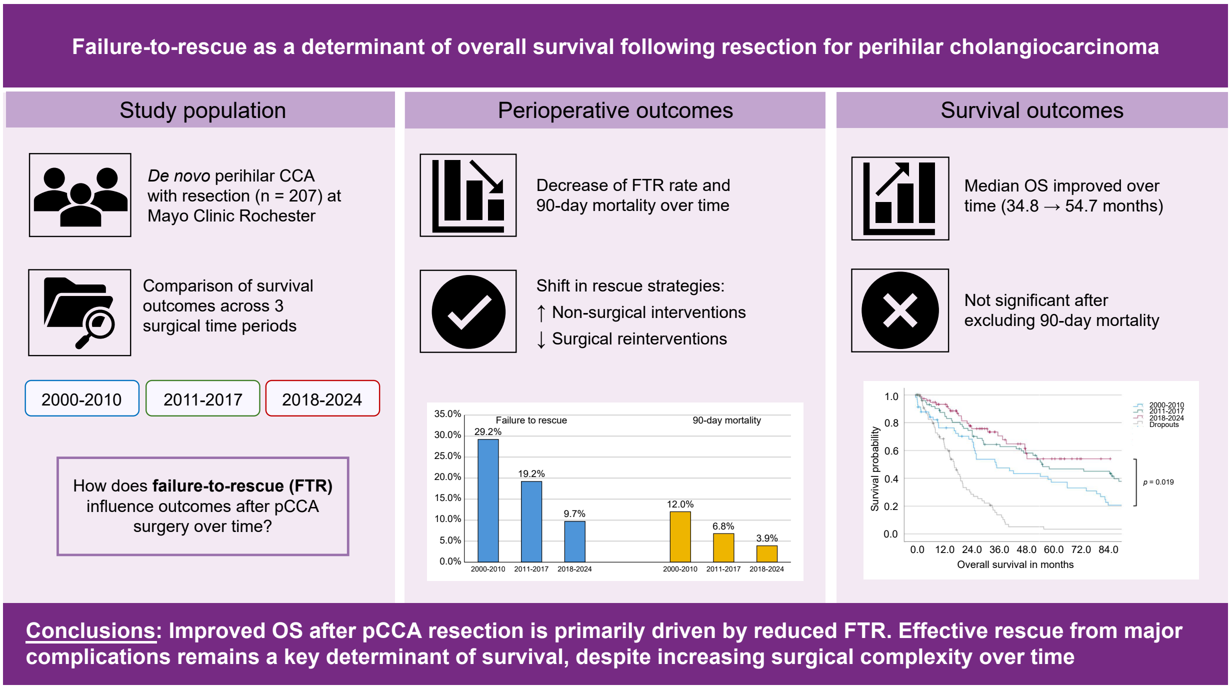

Highlights:

- A marked reduction in FTR rates was the main driver of improved survival in resected *de novo* pCCA.
- Rescue strategy use increased over time, with a notable shift from surgical to non-operative interventions.
- Adjuvant chemotherapy contributed to OS improvements, but showed limited benefit after adjusting for early post-operative mortality.
- Fewer exploratory procedures and dropouts reflect improved preoperative diagnostics and patient selection.

Impact and implications:

While OS in pCCA has improved over recent decades, it remains a malignancy of high surgical complexity and perioperative risk. Our study identified the reduction in FTR as the key determinant of improved outcomes, driven by increased use of rescue strategies and a shift from surgical to non-operative interventions. Although adjuvant therapy became more common over time and contributed to survival gains, its benefit diminished after adjusting for early postoperative mortality. Meanwhile, the declining rate of dropouts further reflects advances in diagnostics and patient selection, emphasizing the importance of multidisciplinary, centralized care in pCCA management.

https://doi.org/10.1016/j.jhepr.2025.101615
© 2025 The Author(s). Published by Elsevier B.V. on behalf of European Association for the Study of the Liver (EASL). This is an open access article under the CC BY license (http://creativecommons.org/licenses/by/4.0/). JHEP Reports, 2025, 7, 1–13

JHEP|Reports

Failure-to-rescue as a determinant of overall survival following resection for perihilar cholangiocarcinoma

Yawen Dong[1], Zhihao Li[2], Vanja Podrascanin[1], John E. Eaton[3], Sumera I. Ilyas[3,4], Gregory J. Gores[3], Susanne G. Warner[1], David M. Nagorney[1], Rory L. Smoot[1,5], Patrick P. Starlinger[1,6,*]

JHEP Reports **2025**. vol. 7 | 1–13

Check for updates

Background & Aims: Perihilar cholangiocarcinoma (pCCA) is a surgically challenging malignancy associated with a high risk of postoperative complications. However, advances in surgical techniques, perioperative care, and systemic therapy might have improved overall survival (OS) over time. This study investigated the evolving impact of failure-to-rescue (FTR) rates and oncological treatment strategies on outcomes in patients with pCCA after curative surgery.

Methods: Patients undergoing curative-intent resection for pCCA at the Mayo Clinic between 2000 and 2024 were retrospectively reviewed. Clinicopathological features were compared across three surgical eras. OS and recurrence-free survival (RFS) were assessed using Kaplan-Meier analysis, with direct matching applied to account for baseline differences.

Results: Among 207 resected patients, 28% were operated on between 2000 and 2010, 35.3% between 2011 and 2017, and 36.7% between 2018 and 2024. The most recent cohort had higher rates of extended hepatectomy, vascular resection, and adjuvant therapy. Although the rate of major complications increased from 36.2% to 40.8%, FTR decreased substantially, from 29.2% to 9.7% (p = 0.066), with a concurrent reduction in 90-day mortality from 12.1% to 3.9% (p = 0.076). Median OS significantly improved over time (from 34.8 to 54.7 months; not reached in 2018–2024, p = 0.019), although this trend was no longer significant after excluding 90-day mortality (p = 0.081). Patients receiving adjuvant therapy had better OS (p = 0.042), but the benefit diminished when 90-day mortality was excluded.

Conclusions: Improved OS after pCCA resection appears to be primarily driven by reduced FTR, despite greater surgical complexity. Effective perioperative management and the ability to rescue patients from major complications remain the key determinants of survival improvement.

© 2025 The Author(s). Published by Elsevier B.V. on behalf of European Association for the Study of the Liver (EASL). This is an open access article under the CC BY license (http://creativecommons.org/licenses/by/4.0/).

Introduction

Perihilar cholangiocarcinoma (pCCA), a malignancy arising from the bile ducts at the liver hilum, remains one of the most technically challenging entities in hepatobiliary surgery. Its close proximity to crucial vascular structures, such as the portal vein and hepatic artery, necessitates highly complex resections, often involving biliary and vascular reconstruction.[1] As a result, pCCA surgery is associated with substantial perioperative morbidity and mortality, underscoring the need for specialized surgical expertise and a coordinated multidisciplinary approach.

Despite these inherent challenges, significant progress has been made over the past decades.[2] Advances in surgical techniques, perioperative care, and institutional experience have collectively contributed to improved overall survival (OS) following resection.[3] However, the complexity of these procedures continues to predispose patients to serious complications, further highlighting the importance of high-level perioperative management.

In this context, failure-to-rescue (FTR), defined as 90-day mortality among patients who experience major postoperative complications, has emerged as an important quality metric for evaluating multidisciplinary postoperative care.[4,5] Although the predictors of morbidity and mortality after pCCA surgery are well established, the role of FTR and its specific determinants in this setting remain less clearly defined. Lower FTR rates reflect the capacity of an institution to promptly recognize and effectively manage life-threatening complications, thereby improving postoperative outcomes and contributing significantly to increased OS. As such, FTR serves not only as a marker of surgical and critical care quality, but also as a pivotal factor in the long-term success of curative-intent pCCA surgery.[6] Given that surgery for pCCA carries one of the highest risks among all elective oncological procedures, with morbidity rates ranging from 45% to 93% and mortality rates from 6% to 18%, FTR represents a crucial measure of institutional performance and patient outcomes in this high-risk population.[7]

* Corresponding author. Address: Department of Surgery, Division of Hepatobiliary and Pancreatic Surgery, Mayo Clinic, 200 First Street SW, Rochester, MN, USA.
E-mail address: starlinger.patrick@mayo.edu (P.P. Starlinger).
https://doi.org/10.1016/j.jhepr.2025.101615

Parallel to surgical and perioperative improvements, major advances have also occurred in the systemic treatment landscape.[8] The integration of neoadjuvant and adjuvant chemotherapy regimens, most notably following the adoption of capecitabine after the BILCAP trial, has added an important oncological dimension to the treatment of pCCA.[9] Although capecitabine is currently regarded as the standard of care in the adjuvant setting for cholangiocarcinoma (CCA), long-term results from the BILCAP trial indicate that its OS benefit remains modest at best, highlighting the need for large-scale, prospective trials with subgroup-specific analysis to establish more effective adjuvant strategies.

In light of these developments, we aimed to assess the impact of temporal improvements in FTR rates and advances in oncological therapies on long-term survival following curative-intent resection for pCCA at a high-volume center.

Methods

Study design and setting

This was a single-center, retrospective cohort study conducted at the Mayo Clinic Rochester, a high-volume tertiary referral center for hepatobiliary malignancies. The study included all patients who underwent curative-intent liver resection for *de novo* pCCA between January 2000 and January 2024.

Participants

Eligible patients were those with *de novo* pCCA who underwent exploratory laparotomy and subsequent liver resection with or without vascular reconstruction. Patients with primary sclerosing cholangitis (PSC)-associated pCCA, recurrent pCCA, combined hepatocellular carcinoma (HCC)-cholangiocarcinoma (CCA), or those undergoing transplantation were excluded. The cohort was grouped into three surgical eras for temporal analysis: 2000–2010; 2011–2017; and 2018–2024. These time intervals were selected to reflect evolving treatment strategies, particularly the implementation of adjuvant therapies post 2010, and to ensure adequate sample size for subgroup analyses. Matching was performed for relevant comparisons (see below).

Patients were classified as high risk if they presented with one or more of the following features: positive resection margins; lymph node involvement; or advanced tumor (T) stage.

Data sources and variables

Clinical, operative, and pathological data were obtained from institutional electronic medical records and prospectively maintained surgical databases. Variables collected included demographics, comorbidities, Eastern Cooperative Oncology Group (ECOG) performance status, preoperative imaging, tumor characteristics (size, T stage, nodal status, and carbohydrate antigen 19-9 [CA 19-9 levels]), type of resection, vascular involvement, complications, and survival outcomes. Resectability was determined by multidisciplinary evaluation based on imaging and clinical criteria to ensure the feasibility of achieving an R0 resection with an adequate future liver remnant (FLR), absence of distant metastases, and acceptable physiological status. In cases of vascular involvement, resection and reconstruction (portal vein, hepatic artery, or both) were performed using direct anastomosis or grafts. Preoperative portal vein embolization (PVE) was used when the FLR was deemed insufficient. Patients found to have locally advanced or metastatic disease during surgical exploration were classified as dropouts.

Definitions

Liver resections were classified per Brisbane 2000 guidelines into minor (less than three segments) and major (three or more segments) resections. Postoperative complications were graded by the Clavien–Dindo system, with severe morbidity defined as Grade ≥III. Posthepatectomy liver failure (PHLF) was defined as per International Study Group of Liver Surgery (ISGLS) criteria and subclassified as primary (insufficient FLR) or secondary (*e.g.* bile leak, sepsis). FTR was defined as mortality within 90 days following a Grade ≥III complication. Resection margins were assessed using American Joint Committee on Cancer (AJCC) 7th or 8th edition guidelines, as appropriate.

Bias and study size

To minimize selection bias, matched analysis was performed for temporal cohort comparisons using 1:1 nearest neighbor matching. Matching variables included age, sex, ECOG (0 *vs.* >0), tumor size (≤3 cm), nodal status, and CA 19-9 level. The total study population represented all eligible cases over the study period, thus encompassing the entire institutional experience with pCCA resection during this timeframe.

Statistical analysis

Continuous variables are presented as medians with IQRs and were compared using the Mann-Whitney *U* test or Kruskal-Wallis H test, as appropriate. Categorical variables are reported as frequencies and percentages and compared using the Chi-square test or Fisher's exact test. Linear regression was applied to assess trends over time.

OS was defined from the date of diagnosis to death or last follow-up. Recurrence-free survival (RFS) was defined from the date of surgery to tumor recurrence or last follow-up. Kaplan-Meier analysis was used to estimate OS and RFS, with comparisons made using log-rank tests. Competing risk analysis for recurrence was performed using the cumulative incidence function, accounting for non-cancer-related deaths, with group comparisons assessed by Gray's test. To evaluate treatment evolution, interventions used for complication management were analyzed by era. Missing data were infrequent and were omitted from the analysis. This retrospective study did not include a formal sensitivity analysis.

All statistical analyses were conducted using IBM SPSS Statistics version 28.0 (IBM Corp., Armonk, NY, USA) and RStudio version 4.3.1 (Posit PBC, Boston, MA, USA). A two-sided $p < 0.05$ was considered statistically significant.

Ethical approval

This study was approved by the Mayo Clinic Institutional Review Board (#22-011911), with informed consent waived due to the retrospective nature of the study. Data were handled in compliance with institutional and federal guidelines for patient confidentiality.

Results

Baseline characteristics and clinicopathological parameters

In total, 207 patients with pathologically confirmed pCCA underwent curative-intent resection at the Mayo Clinic Rochester between 2000 and 2024. Patients were stratified into three cohorts based on surgical era: 2000–2010 (n = 58; 28.0%), 2011–2017 (n = 73; 35.3%), and 2018–2024 (n = 76; 36.7%). Baseline demographics, including sex, age, BMI, and ECOG performance status, were comparable across groups. However, significant differences were noted in preoperative laboratory values (bilirubin, aspartate-aminotransferase-platelet-ratio-index [APRI], albumin–bilirubin score [ALBI] score, international normalized ratio [INR], and albumin). While Bismuth–Corlette classification was similarly distributed, there was a non-significant trend toward more type IV resections in the 2018–2024 cohort (Table 1). Preoperative biliary drainage was most common in the most recent cohort (92.1%), predominantly via endoscopic retrograde cholangiopancreatography (ERCP), and PVE was also performed more frequently in this group (21.1%).

Time period also correlated with differences in clinicopathological and perioperative factors, including extent of liver resection, frequency of vascular reconstruction, tumor size, histological grade, resection margin status, hospital stay, and use of systemic therapy. Notably, a continuous increase in the use of neoadjuvant and adjuvant systemic therapy was observed over time. Furthermore, the most recent cohort showed a higher rate of extended hepatectomy and vascular resections (Table 2).

Temporal trends in major complications, 90-day mortality, FTR rates, and use of rescue strategies

Table 3 summarizes major complications (Clavien–Dindo ≥III), 90-day mortality, and FTR rates across surgical time periods. The most common complications were PHLF, bile leak, and cardiorespiratory events. Although the overall incidence of major complications remained stable over time (p = 0.714), FTR rates improved markedly, declining from 29.2% (2000–2010) to 19.2% (2011–2017), and 9.7% (2018–2024) (p = 0.076; Fig. 1A).

Subgroup analysis by vascular resection and surgical era showed similar complication rates across cohorts. Of note, 90-day mortality in patients with vascular resection decreased more than three-fold in the most recent cohort. Correspondingly, FTR dropped from 33.3% in earlier cohorts to 7.7%, despite a 25% PHLF rate in this subgroup. Causes of FTR within 90 days were primary and secondary PHLF, sepsis, thromboembolic events, and hemorrhage (Table S1).

In parallel with the decline in FTR rates, there was a marked increase in the use of rescue interventions for major

Table 1. Baseline characteristics of all patients with resected pCCA stratified by surgical eras (2000–2010 *vs.* 2011–2017 *vs.* 2018–2024).

	Surgical era			*p* value			
**Parameter**	**2000–2010 (Group A); n = 58**	**2011–2017 (Group B); n = 73**	**2018–2024 (Group C); n = 76**	**A *vs.* B *vs.* C**	**A *vs.* B**	**A *vs.* C**	**B *vs.* C**
Sex				0.507	0.277	0.778	0.384
Male	38 (65.5)	41 (56.2)	48 (63.2)				
Female	20 (34.5)	32 (43.8)	28 (36.8)				
Age (years)	66.5 (54.8–73.0)	62.0 (55.8–68.0)	68.0 (57.5–73.0)	0.191	0.296	0.729	0.054
BMI (kg/m^2)	26.0 (22.9–30.3)	25.3 (23.4–30.2)	25.5 (22.6–28.8)	0.197	0.674	0.078	0.212
ECOG				0.226	0.659	0.090	0.217
0	39 (67.2)	53 (72.6)	61 (80.3)				
>0	19 (32.8)	20 (27.4)	15 19.7)				
Bilirubin (mg/dl)	2.0 (0.8–5.1)	1.4 (0.6–3.9)	0.8 (0.5–1.6)	**<0.001**	**0.048**	**<0.001**	**0.042**
APRI + ALBI	-1.80 (-2.33 to -1.14)	-1.75 (-2.38 to -1.30)	-2.26 (-2.63 to -1.87)	**0.001**	0.247	**<0.001**	**0.010**
Prothrombin time (sec)	9.6 (9.1–10.2)	12.1 (11.3–12.8)	11.7 (11.3–12.6)	**<0.001**	**<0.001**	**<0.001**	0.458
Platelets (x10^9/L)	252.0 (205.0–314.0)	266.0 (200.0–334.0)	251.0 (196.0–307.0)	0.650	0.899	0.432	0.428
INR	1.0 (0.9–1.0)	1.0 (1.0–1.1)	1.1 (1.0–1.2)	**<0.001**	0.201	**<0.001**	**<0.001**
AST (U/L)	65 (43–99)	66 (43–84)	51 (34–81)	**0.015**	0.456	**0.008**	**0.029**
ALT (U/L)	79 (56–145)	75 (56–118)	53 (33–108)	**0.009**	0.245	**0.003**	**0.048**
Albumin (g/dl)	3.9 (3.6–4.2)	3.9 (3.6–4.2)	4.1 (3.8–4.3)	**0.023**	0.537	**0.012**	**0.036**
CA19-9 (U/ml)	146 (27–377)	93 (37–305)	63 (19–389)	0.520	0.593	0.273	0.492
Bismuth–Corlette				0.449	0.448	0.559	0.233
I	1 (1.7)	2 (2.7)	2 (2.6)				
II	1 (1.7)	1 (1.4)	9 (11.8)				
IIIa	28 (48.3)	28 (38.4)	32 (42.1)				
IIIb	25 (43.1)	40 (54.8)	22 (28.9)				
IV	3 (5.2)	2 (2.7)	11 (14.5)				
PreOP stenting	53 (91.4)	63 (86.3)	70 (92.1)	0.454	0.365	0.879	0.253
ERCP	48 (82.8)	54 (74.0)	69 (90.8)				
PTCD	5 (8.6)	9 (12.3)	1 (1.3)				
PVE	2 (3.4)	6 (8.2)	16 (21.1)	**0.004**	0.257	**0.003**	**0.027**
Time from diagnosis to surgery (days)	24 (14–40)	41 (23–61)	63 (32–112)	**<0.001**	**0.003**	**<0.001**	**0.002**

Patients were stratified by surgical eras: Group A (2000–2010), Group B (2011–2017), and Group C (2018–2024). Continuous variables are presented as medians with IQRs and were compared using the Kruskal-Wallis H test for overall group comparisons (A *vs.* B *vs.* C), and the Mann-Whitney *U* test for pairwise comparisons. Categorical variables are reported as n (%) and were compared using the Chi-square test or Fisher's exact test, as appropriate; p <0.05 was considered statistically significant. ALBI, albumin–bilirubin score; ALT, alanine aminotransferase; APRI, aspartate aminotransferase to platelet ratio Index; AST, aspartate aminotransferase; CA19-9, carbohydrate antigen 19-9; ECOG, Eastern Cooperative Oncology Group; ERCP, endoscopic retrograde cholangiopancreatography; INR, international normalized ratio; pCCA, perihilar cholangiocarcinoma; PTCD, percutaneous transhepatic cholangiographic drainage; PVE, portal vein embolization.

Table 2. Clinicopathological parameters of all patients with resected pCCA stratified by surgical era (2000–2010 *vs.* 2011–2017 *vs.* 2018–2024).

	Surgical era			*p* value			
**Parameter**	**2000–2010 (Group A); n = 58**	**2011–2017 (Group B); n = 73**	**2018–2024 (Group C); n = 76**	**A *vs.* B *vs.* C**	**A *vs.* B**	**A *vs.* C**	**B *vs.* C**
Type of liver resection				**<0.001**	**0.037**	**<0.001**	**0.007**
Right hepatectomy	27 (46.6)	19 (26.0)	19 (25.0)				
Extended right hepatectomy	4 (6.9)	10 (13.7)	24 (31.6)				
Left hepatectomy	24 (41.4)	35 (47.9)	17 (22.4)				
Extended left hepatectomy	1 (1.7)	8 (11.0)	15 (19.7)				
Central hepatectomy	2 (3.4)	1 (1.4)	1 (1.3)				
Vascular reconstruction	6 (10.3)	11 (15.1)	20 (26.3)	**0.042**	0.424	**0.021**	0.091
TNM stage (AJCC 8th edition)				0.156	0.074	0.053	0.703
Stage 0	0 (0)	0 (0)	1 (1.3)				
Stage 1	4 (6.9)	9 (12.3)	13 (17.1)				
Stage 2	15 (25.9)	27 (37.0)	26 (34.2)				
Stage 3	38 (65.5)	35 (47.9)	30 (39.5)				
Stage 4	1 (1.7)	2 (2.7)	6 (7.9)				
Tumor size (cm)	2.7 (2.2–3.1)	2.6 (2.3–3.8)	3.1 (2.3–4.0)	**0.013**	0.201	**0.009**	0.291
Lymph node status				0.062	0.916	0.155	0.196
N0	30 (51.7)	39 (53.4)	33 (43.4)				
N1	28 (48.3)	30 (41.1)	36 (47.4)				
N2	0 (0)	0 (0)	3 (3.9)				
Nx	0 (0)	4 (5.5)	4 (5.3)				
Histological grade				**<0.001**	**<0.001**	**<0.001**	0.610
G1	3 (5.2)	12 (16.4)	9 (11.8)				
G2	10 (17.2)	39 (53.4)	48 (63.2)				
G3	42 (72.4)	19 (26.0)	18 (23.7)				
G4	3 (5.2)	3 (4.1)	0 (0)				
NA	0 (0)	0 (0)	1 (1.3)				
Resection margin				**0.003**	0.188	**0.001**	**0.031**
R0	53 (91.4)	61 (83.6)	52 (68.4)				
R1	5 (8.6)	12 (16.4)	24 (31.6)				
PHLF grade B/C	9 (15.5)	13 (17.8)	15 (19.7)	0.819	0.728	0.528	0.763
Estimated blood loss (ml)	700 (500–800)	750 (350–1,500)	600 (300–1,000)	0.168	0.631	0.411	0.097
PostOP stay (days)	9 (6–14)	6 (5–9)	7 (4–12)	**0.037**	**0.003**	**0.042**	0.437
Morbidity				0.712	0.203	0.369	0.700
Clavien–Dindo 0	23 (39.7)	12 (16.4)	17 (22.4)				
Clavien–Dindo I	4 (6.9)	13 (17.8)	13 (17.2)				
Clavien–Dindo II	8 (13.8)	22 (30.2)	15 (19.7)				
Clavien–Dindo III	10 (17.2)	11 (15.1)	21 (27.6)				
Clavien–Dindo IV	8 (13.8)	10 (13.7)	7 (9.2)				
Clavien–Dindo V	3 (5.1)	5 (6.8)	3 (3.9)				
NA	2 (3.5)	0 (0)	0 (0)				
Major complications (Clavien–Dindo ≥III)	21 (36.2)	26 (35.6)	31 (40.8)	0.714	0.450	0.879	0.516
90-day mortality	7 (12.1)	5 (6.8)	3 (3.9)	0.076	0.304	0.076	0.432
Neoadjuvant therapy				**0.045**	0.108	**0.013**	0.281
Yes	2 (3.4)	8 (11.0)	13 (17.1)				
No	56 (96.6)	65 (89.0)	63 (82.9)				
Adjuvant therapy				**<0.001**	**0.011**	**<0.001**	**0.002**
Yes	13 (22.4)	31 (42.5)	52 (68.4)				
No	45 (77.6)	42 (57.5)	24 (31.6)				
Time to adjuvant therapy (days)	63.0 (35.0–84.0)	55.0 (49.5–68.5)	65.0 (53.5–78.0)	0.293	0.952	0.497	0.114
Type of adjuvant therapy				**<0.001**	**0.001**	**<0.001**	**<0.001**
Gemcitabine-based regimens	3 (23.1)	20 (64.6)	16 (30.8)				
Capecitabine monotherapy	0 (0)	4 (12.9)	24 (46.2)				
Concomitant chemoradiation	8 (61.5)	4 (12.9)	10 (19.2)				
Chemoradiation followed by chemotherapy	1 (7.7)	1 (3.2)	2 (3.8)				
CAPOX (capecitabine/oxaliplatin)	0 (0)	1 (3.2)	0 (0)				
NA	1 (7.7)	1 (3.2)	0 (0)				
Follow-up time (months)	25.3 (8.6–78.4)	46.3 (18.1–91.6)	24.4 (12.0–45.9)	**0.004**	0.191	0.244	**<0.001**
Recurrence	21 (36.2)	36 (49.3)	30 (39.5)	0.232	0.140	0.881	0.147
Location of recurrence				0.490	0.229	0.690	0.464
Local	7 (12.1)	12 (16.4)	9 (11.8)				
Distant	14 (24.1)	19 (26.0)	19 (25)				

Continuous variables are presented as medians with IQRs and compared using the Kruskal-Wallis H test for overall group comparisons (A *vs.* B *vs.* C), and the Mann-Whitney *U* test for pairwise comparisons. Categorical variables are reported n (%) and were compared using the Chi-square test or Fisher's exact test, as appropriate; $p < 0.05$ was considered statistically significant. AJCC, American Joint Committee on Cancer; NA, not available; pCCA, perihilar cholangiocarcinoma; PHLF, posthepatectomy liver failure.

Table 3. Incidence of major complications (Clavien–Dindo ≥ III), 90-day mortality, and FTR rates in the vascular, non-vascular, and total cohorts, stratified by surgical era (2000–2010 *vs.* 2011–2017 *vs.* 2018–2024).

		Surgical group			*p* value			
**All resected pCCA cases (N = 207)**		**2000–2010 (Group A); n = 58**	**2011–2017 (Group B); n = 73**	**2018–2024 (Group C); n = 76**	**A *vs.* B *vs.* C**	**A *vs.* B**	**A *vs.* C**	**B *vs.* C**
Clavien–Dindo ≥III	81 (39.1)	24 (41.3)	26 (35.6)	31 (40.8)	0.714	0.450	0.879	0.516
Bile leak	17 (8.2)	2 (3.4)	6 (8.2)	9 (11.8)				
Posthepatectomy liver failure	34 (16.4)	11 (19.0)	9 (12.3)	14 (18.4)				
Vascular complications (arterial/portal vein thrombosis)	3 (1.4)	1 (1.7)	1 (1.4)	1 (1.3)				
Posthepatectomy hemorrhage	8 (3.9)	2 (3.4)	2 (2.7)	4 (5.3)				
Intraabdominal abscess	8 (3.9)	3 (5.2)	3 (4.1)	2 (2.6)				
Cardiorespiratory complications (pleural effusion requiring thoracocentesis, tachycardic atrial fibrillation requiring ICU management, sudden cardiac arrest, etc.)	11 (5.3%)	5 (8.6)	5 (6.8)	1 (1.3)				
90-day mortality	15 (7.2)	7 (12.0)	5 (6.8)	3 (3.9)	0.076	0.304	0.076	0.432
FTR rate (%)	**18.5**	**29.2**	**19.2**	**9.7**	0.066	0.416	0.066	0.301
**pCCA without vascular resection (n = 170)**		**2000–2010 (Group A); n = 52**	**2011–2017 (Group B); n = 62**	**2018–2024 (Group C); n = 56**	**A *vs.* B *vs.* C**	**A *vs.* B**	**A *vs.* C**	**B *vs.* C**
Clavien–Dindo ≥III	62 (36.5)	21 (40.4)	23 (37.1)	18 (32.1)	0.668	0.719	0.373	0.573
Bile leak	12 (7.1)	1 (1.9)	5 (8.1)	6 (10.7)				
Posthepatectomy liver failure	27 (15.9)	10 (19.2)	8 (12.9)	9 (16.1)				
Posthepatectomy hemorrhage	6 (3.5)	2 (3.8)	2 (3.2)	2 (3.6)				
Intraabdominal abscess	6 (3.5)	3 (5.8)	3 (4.8)	0 (0)				
Cardiorespiratory complications (pleural effusion requiring thoracocentesis, tachycardic atrial fibrillation requiring ICU management, sudden cardiac arrest, etc.)	11 (6.5)	5 (9.7)	5 (8.1)	1 (1.8)				
90-day mortality	12 (7.1)	6 (11.5)	4 (6.4)	2 (3.6)	0.264	0.339	0.114	0.477
FTR rate (%)	**19.4**	**28.6**	**17.4**	**11.1**	0.168	0.382	0.184	0.577
**pCCA with vascular resection (n = 37)**		**2000–2010 (Group A); n = 6**	**2011–2017 (Group B); n = 11**	**2018–2024 (Group C); n = 20**	**A *vs.* B *vs.* C**	**A *vs.* B**	**A *vs.* C**	**B *vs.* C**
Clavien–Dindo ≥ III	19 (51.4)	3 (50)	3 (27.3)	13 (65.0)	0.124	0.210	0.835	**0.044**
Bile leak	5 (13.6%)	1 (16.7)	1 (9.1)	3 (15)				
Posthepatectomy liver failure	7 (18.9)	1 (16.7)	1 (9.1)	5 (25)				
Vascular complications (arterial/portal vein thrombosis)	3 (8.1)	1 (16.7)	1 (9.1)	1 (5)				
Posthepatectomy hemorrhage	2 (5.4)	0 (0)	0 (0)	2 (10)				
Intraabdominal abscess	2 (5.4)	0 (0)	0 (0)	2 (10)				
90-day mortality	3 (8.1)	1 (16.7)	1 (9.1)	1 (5.0)	0.649	0.643	0.347	0.657
FTR rate (%)	**15.8**	**33.3**	**33.3**	**7.7**	0.198	0.999	0.241	0.241

Continuous variables are presented as medians with IQRs and were compared using the Kruskal-Wallis H test for overall group comparisons (A *vs.* B *vs.* C), and the Mann-Whitney *U* test for pairwise comparisons. Categorical variables are reported as n (%) and were compared using the Chi-square test or Fisher's exact test, as appropriate; $p < 0.05$ was considered statistically significant. FTR rate = $\frac{\textit{Number of deaths after major complications}}{\textit{Total number of patients with major complications}}$. FTR, failure-to-rescue; ICU, intensive care unit; pCCA, perihilar cholangiocarcinoma.

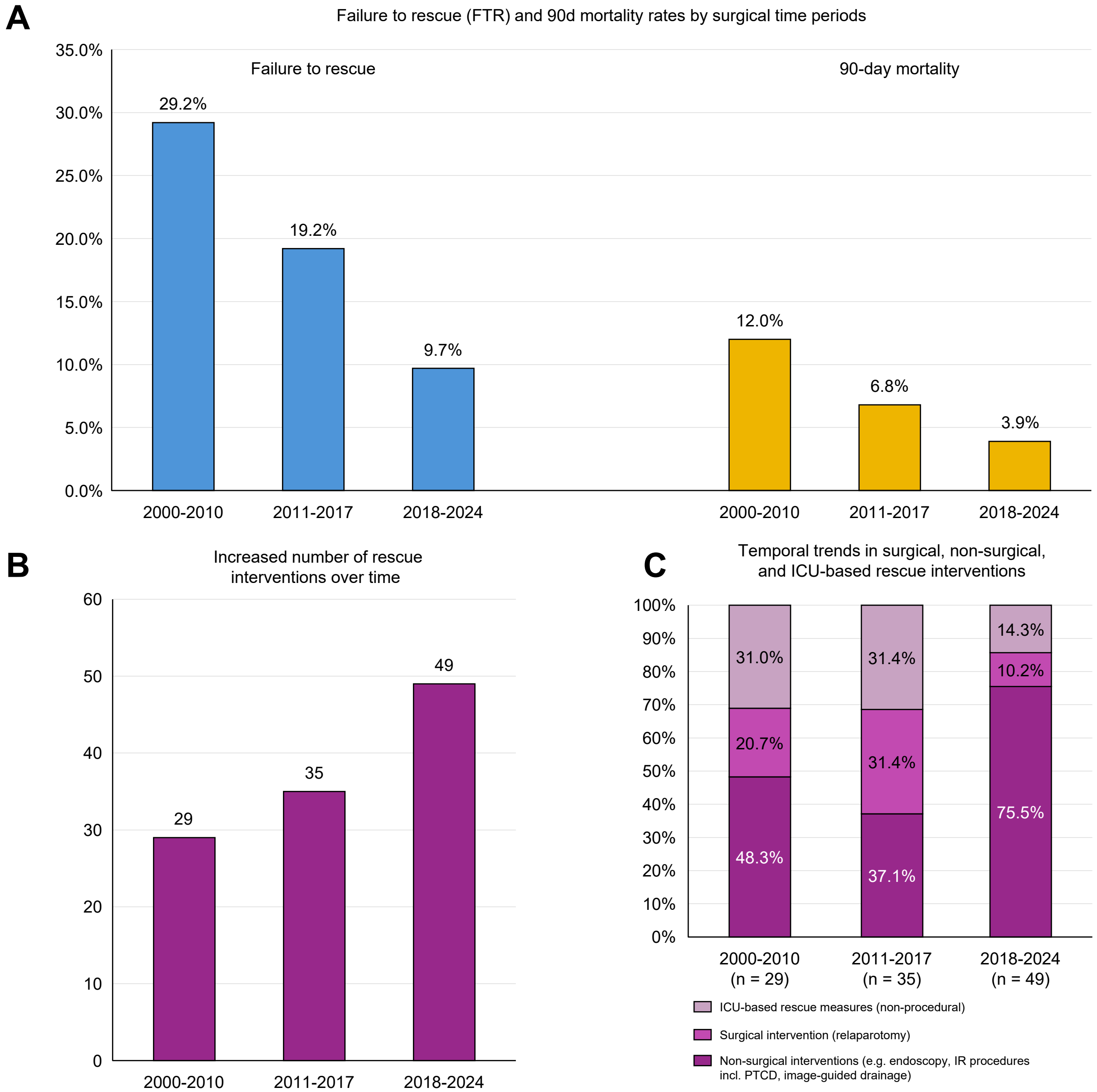

Fig. 1. Temporal trends in FTR, 90-day mortality, and rescue strategies. (A) FTR and 90-day mortality rates stratified by surgical time periods (2000–2010, 2011–2017, and 2018–2024). (B) Temporal trends in rescue interventions following major postoperative complications. (C) Distribution of rescue strategies over time, highlighting an increase in non-surgical interventions, such as endoscopy and IR-guided procedures, along with a corresponding decline in surgical reinterventions during the most recent era (2018–2024). FTR, failure-to-rescue; ICU, intensive care unit; IR, interventional radiology; PTCD, percutaneous transhepatic cholangiographic drainage.

postoperative complications over time (Fig. 1B,C; Table S2). Although the use of endoscopic and intensive care unit (ICU)-based measures remained relatively stable, the most pronounced increases were observed in image-guided drainage (*e.g.* for abscesses or fluid collections), percutaneous transhepatic biliary drainage (PTCD) for bile leak management, and interventional radiology (IR)-guided vascular procedures, such as embolization or portal vein stenting. These interventions, which were rarely utilized in the earliest cohort (2000–2010), became substantially more common in the most recent era (2018–2024), reflecting the increasing integration of multidisciplinary and minimally invasive approaches into postoperative care. This temporal evolution in complication management likely contributed to the improved rescue rates and declining FTR observed over time.

Comparison of rescued group versus FTR group

A subgroup analysis was conducted among patients who experienced major complications, comparing the rescued group with the FTR group. Interestingly, patients in the FTR group had a higher BMI and more severely impaired preoperative liver function, as reflected by a significantly abnormal APRI + ALBI score (p = 0.023) and lower albumin levels (p = 0.038). Furthermore, a significant difference in the extent of

liver resection was observed ($p = 0.033$), with left hepatectomy most frequently performed in the FTR group (53.3%). By contrast, no significant differences were found in other key variables, including use of neoadjuvant therapy, incidence of PHLF, tumor stage, Bismuth–Corlette classification, or vascular reconstruction (Table S3).

Temporal improvement of OS in all-comers and matched cohorts

Median follow-up was 25.3 months (2000–2010), 46.3 months (2011–2017), and 24.4 months (2018–2024). Kaplan-Meier analysis of all patients with resected pCCA demonstrated a significant improvement in OS over time ($p = 0.019$, Fig. 2A). Median OS increased from 34.8 months (95% CI, 19.1–50.5) in 2000–2010 to 54.7 months (95% CI, 24.7–84.8) in 2011–2017, while the median was not reached in 2018–2024. Patients who underwent exploration without resection had significantly lower OS (16.8 months, 95% CI, 13.1–20.5), with the number of dropouts declining from 46 (2000–2010) to 11 (2018–2024; Fig. S1).

Excluding 90-day mortality, OS showed a trend toward improvement across surgical eras ($p = 0.081$), increasing from 42.3 months (95% CI, 14.1–70.5) in 2000–2010 to 72.4 months (95% CI, 36.8–108.0) in 2011–2017, with the median not reached in 2018–2024 (Fig. 2B).

To account for biological differences between patients, 1:1 matching was performed based on age, ECOG, tumor size >3 cm, nodal status, and preoperative CA 19-9 (n = 49 per group). In the matched population, OS significantly improved over time ($p = 0.020$): 35.1 months (95% CI, 15.1–55.2) in 2000–2010, 54.3 months (95% CI, 17.3–91.3) in 2011–2017, and not reached in 2018–2024 (Fig. 2C). Excluding 90-day mortality, OS continued to favor the most recent cohort, although without statistical significance ($p = 0.068$; Fig. 2D).

A

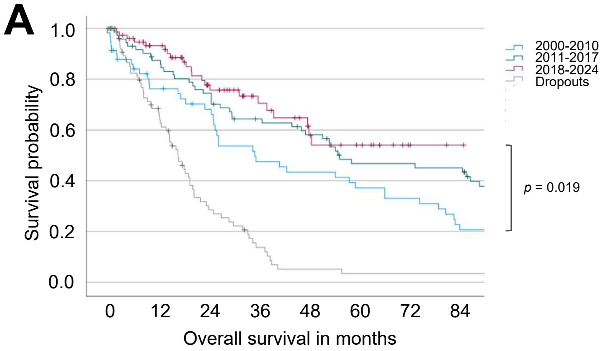

No. at risk								
2000-2010	58	39	33	23	21	18	16	10
2011-2017	73	61	52	42	37	28	28	24
2018-2024	76	62	39	25	15	10	2	NR
Dropouts	81	48	18	8	3	2	2	2

B

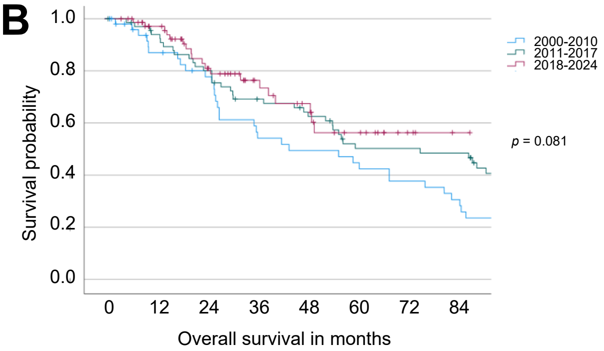

No. at risk								
2000-2010	51	39	33	23	21	18	16	10
2011-2017	68	61	52	42	37	28	28	24
2018-2024	73	62	39	25	15	10	2	NR

C

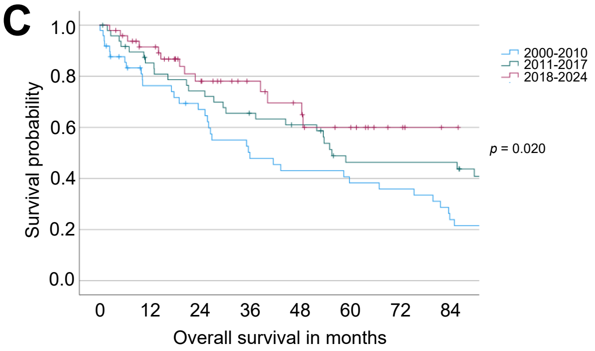

No. at risk								
2000-2010	49	33	28	20	18	16	15	9
2011-2017	49	39	34	29	26	18	18	16
2018-2024	49	40	25	19	11	8	2	0

D

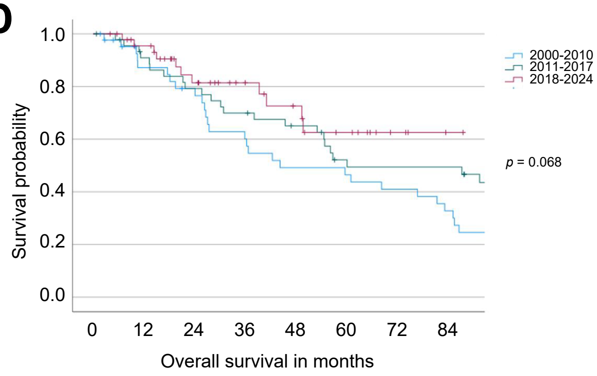

No. at risk								
2000-2010	43	33	28	20	18	16	15	9
2011-2017	46	39	34	29	26	18	18	16
2018-2024	47	40	25	19	11	8	2	0

Fig. 2. OS analyses in patients with resected pCCA across surgical eras. (A) Kaplan-Meier curve demonstrating OS of patients with resected pCCA and dropouts stratified by surgical eras. (B) OS analysis of all patients with resected pCCA stratified by time period of surgery with exclusion of 90-day mortality cases. (C) OS analysis of patients with resected pCCA after 1:1 direct matching balanced for the parameters age, ECOG, tumor size >3 cm, LN status, CA19-9, stratified by surgical eras. (D) Kaplan-Meier curve demonstrating OS of matched patients stratified by surgical eras excluding 90-day mortality cases. Data were analyzed using Kaplan-Meier survival estimates and log-rank tests; p <0.05 was considered statistically significant. CA19-9, carbohydrate antigen 19-9; ECOG, Eastern Cooperative Oncology Group; LN, lymph node; NR, not reached; OS, overall survival; pCCA, perihilar cholangiocarcinoma.

RFS did not differ significantly between eras (p = 0.560; Fig. 3A). Median RFS was 60.1 months (95% CI, 0.0–146.4) in 2000–2010, 52.2 months (95% CI, 12.1–92.3) in 2011–2017, and 29.0 months (95% CI, 0.6–57.4) in 2018–2024. A focused 2-year RFS analysis, accounting for shorter follow-up in recent cases, showed comparable RFS across cohorts (Fig. 3B).

Competing risk analysis treating recurrence as the primary event and non-cancer deaths as competing events revealed a significantly different cumulative incidence of recurrence in unmatched patients (p = 0.008; Fig. 3C), highest in 2018–2024 and lowest in 2000–2010. This difference was not observed in the matched cohorts (p = 0.740; Fig. 3D).

Impact of systemic therapy on survival outcomes

A continuous increase in the use of neoadjuvant and adjuvant systemic therapy was observed over time (Fig. 4A). Neoadjuvant therapy was administered in 11.1% (n = 23) of all patients with resected disease, whereas 46.4% (n = 96) received adjuvant therapy. To assess the potential impact, particularly of adjuvant therapy after 2010, patients were grouped into a recent cohort (combining 2011–2017 and 2018–2024) and a historical cohort (2000–2010). Median OS was significantly longer in the recent cohort (57.5 months, 95% CI, 30.0–85.0) compared with the historical group (34.8 months, 95% CI, 19.1–50.5; p = 0.007; Fig. S2).

Further analysis stratified patients by adjuvant therapy use (Table S4). Median OS was significantly longer in those who received adjuvant treatment (57.0 *vs.* 52.4 months; p = 0.042; Fig. 4B). However, in matched subgroups, adjusted for age, ECOG, tumor size, lymph node status, and CA 19-9, and after excluding 90-day mortality, the survival benefit was attenuated and no longer statistically significant (p = 0.117 and p = 0.711, respectively; Fig. 4C,D).

Value of adjuvant therapy stratified by major complications and high-risk pathology

To further determine the impact of adjuvant therapy, a subgroup analysis was performed stratifying patients by adjuvant

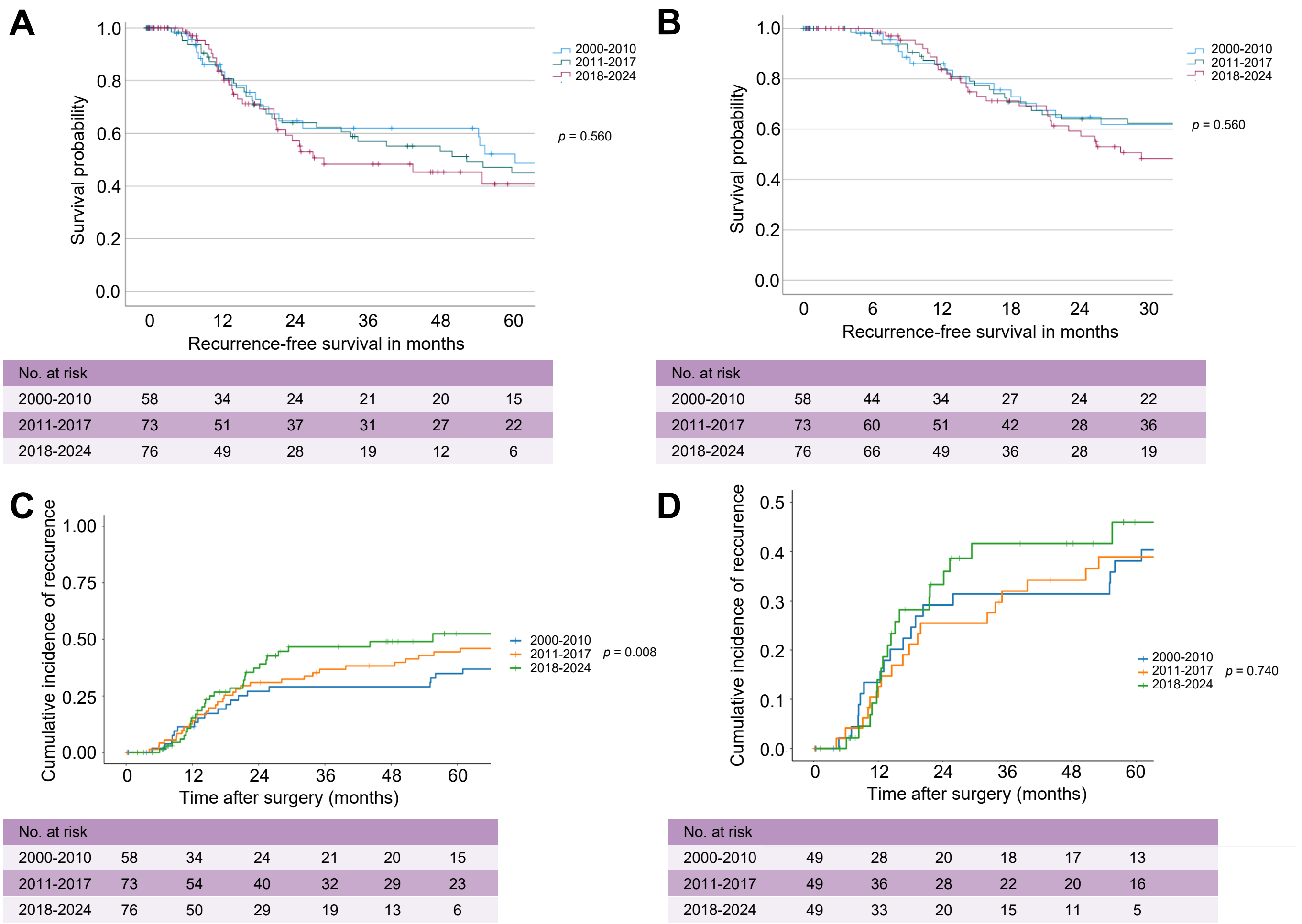

A – No. at risk

No. at risk	0	12	24	36	48	60
2000-2010	58	34	24	21	20	15
2011-2017	73	51	37	31	27	22
2018-2024	76	49	28	19	12	6

B – No. at risk

No. at risk	0	6	12	18	24	30
2000-2010	58	44	34	27	24	22
2011-2017	73	60	51	42	28	36
2018-2024	76	66	49	36	28	19

C – No. at risk

No. at risk	0	12	24	36	48	60
2000-2010	58	34	24	21	20	15
2011-2017	73	54	40	32	29	23
2018-2024	76	50	29	19	13	6

D – No. at risk

No. at risk	0	12	24	36	48	60
2000-2010	49	28	20	18	17	13
2011-2017	49	36	28	22	20	16
2018-2024	49	33	20	15	11	5

Fig. 3. RFS in all patients with resected pCCA, stratified by surgical eras. (A) Overall RFS. (B) RFS limited to 2 years to account for the shorter follow-up period in the most recent cohort. (C,D) RFS and competing risk analysis for (C) all-comers and (D) after direct matching. Criteria for direct matching: age, ECOG, tumor size >3 cm, LN status, and preoperative CA19-9. RFS was analyzed using Kaplan-Meier estimates and log-rank tests for (A,B). For (C,D), competing risk analysis was performed using the cumulative incidence function, with Gray's test applied to assess differences between groups; p <0.05 was considered statistically significant. CA19-9, carbohydrate antigen 19-9; ECOG, Eastern Cooperative Oncology Group; LN, lymph node; pCCA, perihilar cholangiocarcinoma; RFS, recurrence-free survival.

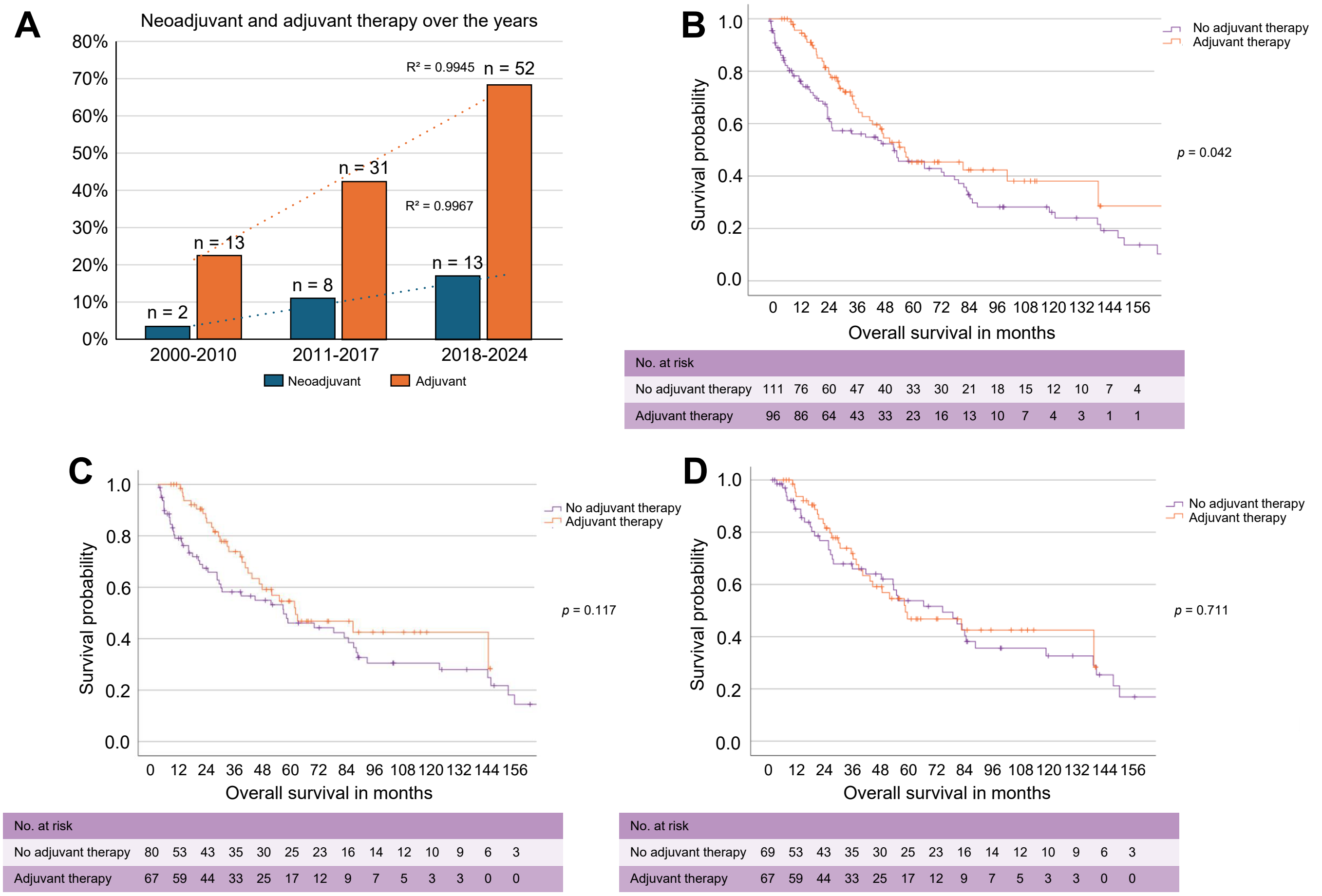

Fig. 4. Impact of adjuvant therapy on OS. (A) Increasing use of neoadjuvant and adjuvant therapy in patients with resected pCCA across surgical eras (2000–2010, 2011–2017, and 2018–2024). Linear regression was performed to illustrate temporal trends, with corresponding R^2 values shown. (B) Stratification of patients based on the actual receipt of adjuvant therapy, evaluating its association with OS. (C,D) Evaluation of the impact of adjuvant therapy on OS in patients with resected pCCA stratified by (C) receipt of adjuvant therapy after direct matching and (D) additionally excluding 90-day mortality cases. OS was analyzed using Kaplan-Meier estimates and log-rank tests; *p* <0.05 was considered statistically significant. CTx, chemotherapy; OS, overall survival; pCCA, perihilar cholangiocarcinoma.

therapy, major postoperative complications, and high-risk pathological features (Table S5). Among patients who did not receive adjuvant therapy, those with high-risk features and major complications had the poorest prognosis (median OS, 10.0 months; 95% CI, 2.0–18.0), compared with high-risk patients without major complications (45.6 months; 95% CI, 26.4–64.8) and patients who received adjuvant therapy (57.0 months; 95% CI, 27.6–86.4; *p* <0.001; Fig. 5A,B). This trend persisted when the analysis was limited to high-risk patients, with significant OS differences based on adjuvant therapy and complication status (*p* <0.001; Fig. 5C). By contrast, no significant OS differences were observed within the low-risk cohort (*p* = 0.445; Fig. S3). Notably, in high-risk patients without major complications, OS was comparable regardless of adjuvant therapy use (*p* = 0.289; Fig. 5D).

Discussion

In this retrospective study, which included 207 patients who underwent curative-intent resection for *de novo* pCCA between 2000 and 2024, we observed a clear improvement in OS over time. This survival benefit appeared to be primarily driven by two major contributors: first and predominantly, a pronounced reduction in FTR rates among patients with major postoperative complications, and likely also second, the increasing use of systemic therapies, including both neoadjuvant and adjuvant approaches, as newer treatment options for CCA have become available. Alongside these factors, improved patient selection and preoperative optimization strategies also contributed to the overall progress in surgical outcomes. Notably, whereas the incidence of major complications rose in the most recent cohort, largely as a result of an increase in technically demanding procedures, such as vascular reconstructions, this did not translate into higher perioperative mortality. Instead, the improved capacity to manage complications reflects advances in perioperative care, facilitated by refined surgical techniques and enhanced interdisciplinary collaboration, ultimately contributing to the observed survival benefit and highlighting the importance of both surgical expertise and coordinated perioperative support in achieving favorable outcomes in patients with pCCA.

FTR has emerged as a crucial quality metric in complex surgical care, particularly within the field of Hepato-pancreatobiliary surgery.[10–13] Although FTR has been extensively studied

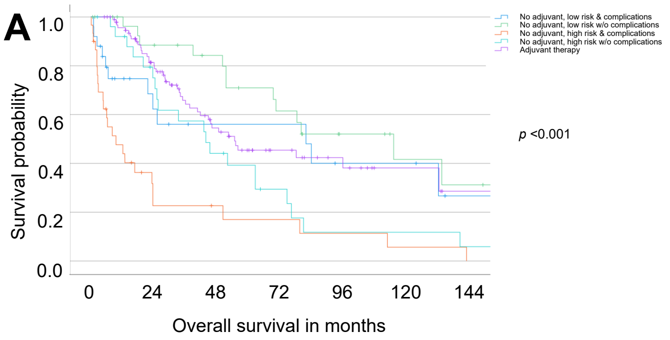

No. at risk							
No adjuvant, low risk & complications	25	11	7	7	5	4	1
No adjuvant, low risk w/o complications	30	34	19	15	9	4	3
No adjuvant, high risk & complications	30	8	4	3	2	1	1
No adjuvant, high risk w/o complications	26	18	10	5	2	2	2
Adjuvant therapy	96	64	32	16	10	4	1

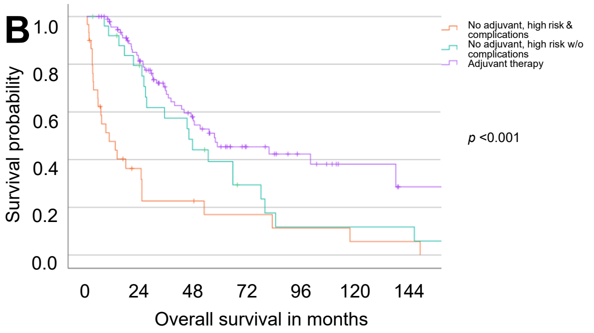

No. at risk							
No adjuvant, high risk & complications	30	8	4	3	2	1	1
No adjuvant, high risk w/o complications	26	18	10	5	2	2	2
Adjuvant therapy	96	64	32	16	10	4	1

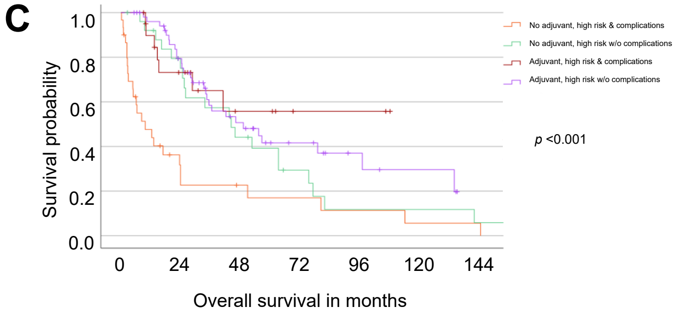

No. at risk							
No adjuvant, high risk & complications	30	8	4	3	2	1	1
No adjuvant, high risk w/o complications	26	18	10	5	2	2	2
Adjuvant, high risk complications	21	12	5	2	2	0	0
Adjuvant, high risk w/o complications	53	37	19	10	5	3	0

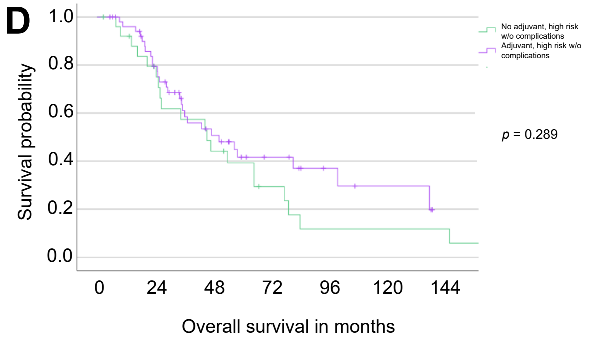

No. at risk							
No adjuvant, high risk & complications	26	18	10	5	2	2	1
No adjuvant, high risk w/o complications	53	37	19	10	5	3	10

Fig. 5. Subgroup analysis of adjuvant *vs.* no adjuvant therapy stratified by major complications and low-/high-risk profile. (A) OS comparison between the no adjuvant therapy cohort stratified by low-/high-risk profile and major complications *vs.* the adjuvant therapy cohort. (B) OS comparison between high-risk profile patients within the no adjuvant therapy cohort stratified by major complications *vs.* the adjuvant therapy cohort. (C) OS comparison in high-risk profile patients stratified by adjuvant therapy and major complications. (D) Subgroup analysis of OS comparing high-risk profile patients without complications stratified by receipt of adjuvant therapy. OS was analyzed using Kaplan-Meier estimates and log-rank tests; p <0.05 was considered statistically significant. OS, overall survival.

in tumor entities, such as HCC and pancreatic cancer, data specific to pCCA remain limited.[4,5,14] The present study offers novel insights into the evolution of OS and FTR rates in patients with resected pCCA treated at a high-volume, single-center institution over the past two decades. By stratifying patients into three cohorts according to surgical era, while accounting for evolving trends in systemic therapy and ensuring balanced case distribution between cohorts, we observed an increased rate of vascular resections and a corresponding rise in major complications in the most recent era. Notably, despite the higher complication burden, the FTR rate decreased substantially over time, to a clinically meaningful degree: FTR dropped from 29% in the earliest cohort to <10% in the most recent. These findings align with previous reports and underscore the importance of focusing on not only preventing complications, but also enhancing the capacity to manage them effectively.[15–17] This led to a noteworthy reduction of 90-day mortality to 3.9% despite 30% of cases requiring vascular resection in the most recent cohort. Therefore, in the complex surgical landscape of pCCA, reducing FTR could represent one of the most impactful strategies to improve overall outcomes after curative-intent resection.

The observed improvement in FTR rates also highlights the growing importance of multidisciplinary collaboration in the management of patients undergoing resection for pCCA. Over the past decades, the routine integration of interventional radiologists, endoscopists, anesthesiologists, and intensivists into perioperative management has profoundly impacted postsurgical outcomes. Timely interventions, such as endoscopic biliary drainage, percutaneous abscess control, vascular embolization, and critical care optimization, have had a crucial role in managing complications when they occur.[4,14] This coordinated approach has not only enhanced complication recognition and response times, but also expanded the range of effective interventions available for managing life-threatening events postoperatively. Indeed, within our study cohort, there was a clear temporal increase in the use of interventional, non-surgical rescue procedures, particularly IR-guided strategies. As such, the reduction in FTR observed in our cohort likely reflects a broader institutional shift toward

team-based, high-acuity care, which has become essential for improving outcomes in complex hepatobiliary surgery. Indeed, the leading cause of major complications across all surgical eras was PHLF.[18,19] Admittedly, this trend might also reflect improvements in postoperative documentation and diagnostic capabilities, facilitating earlier recognition and management of complications. However, these advances alone do not fully account for the observed outcomes. Equally important is the role of thorough preoperative optimization, which directly influences a patient's ability to withstand major surgery and recover from complications. In the present study, the comparison between rescued patients and those who experienced FTR revealed significant differences in key baseline characteristics, including BMI, liver function parameters, such as the APRI + ALBI score and albumin, as well as the extent of surgery.[20] A similar trend supporting the role of improved preoperative optimization was observed when comparing patients across different surgical eras, with lower preoperative bilirubin levels, better APRI + ALBI scores, and a higher frequency of preoperative biliary drainage in the most recent cohort. Furthermore, patient selection appeared to have also improved over time, as evidenced by a considerable reduction in exploratory procedures without resection, reflecting more accurate preoperative staging and better identification of surgical candidates.

In parallel with surgical and perioperative advances, the oncological landscape for CCA has evolved significantly in recent years, driven by the introduction of novel systemic treatment strategies. Notably, the integration of immune checkpoint inhibitors for advanced-stage disease, as shown in the TOPAZ-1 and KEYNOTE-966 trials, and the implementation of adjuvant therapy protocols, including capecitabine based on the BILCAP trial, have marked important milestones in the systemic treatment landscape of CCA.[9,21,22] Although the BILCAP trial did not meet its primary endpoint in the intention-to-treat analysis, a survival benefit was observed in the preplanned sensitivity analysis for both OS and RFS, which ultimately led to the adoption of capecitabine as standard of care in the adjuvant setting.[23] Indeed, in our most recent cohort, most patients received capecitabine postoperatively. However, the efficacy of adjuvant capecitabine remains a subject of ongoing debate, given that the recently published long-term follow-up of the BILCAP trial showed no survival benefit in the pCCA subgroup. Furthermore, the use of systemic therapy in general increased steadily over time in our cohort. Nearly 70% of patients in the most recent group received adjuvant treatment compared with only 22% in the earliest group. Although an initial survival benefit was shown when stratifying by receipt of adjuvant therapy, this difference was no longer significant after excluding 90-day mortality. In addition, among high-risk patients who did not experience major postoperative complications, OS was comparable regardless of adjuvant therapy. These findings suggest that, although systemic therapy might have a contributory role, its impact appears secondary to perioperative factors, most notably, the marked reduction in early postoperative mortality and FTR rates, which more convincingly account for the observed benefit in long-term survival.

Moreover, although the most recent cohort exhibited worse RFS in unadjusted analyses, competing risk analysis with direct matching revealed comparable RFS across time periods. This finding aligns with the growing tendency to operate on more anatomically complex and borderline-resectable cases in recent years, which likely contributed to the observed increase in R1 resection rates. However, this trend should not be interpreted as a decline in surgical quality. Rather, it likely reflects evolving pathological practices, including more meticulous sampling and stricter definitions of margin status, which might have led to reclassification of cases previously deemed R0.[24–26] Supporting this interpretation, local recurrence rates declined over time despite higher R1 rates, suggesting improved oncological control. A likely contributing factor is the increased use of adjuvant therapy: 71% of patients with R1 resections received postoperative treatment, including combined chemoradiotherapy in 53% of cases. These findings suggest that intensified adjuvant strategies helped mitigate the impact of narrower margins, preserving favorable outcomes despite increased technical complexity.

Of note, the substantial proportion of censored cases in the most recent cohort predominantly reflects patients who were alive at the time of last follow-up rather than losses resulting from disease progression or death. Given that the Mayo Clinic serves as a tertiary referral center, many patients resume routine care with local providers after the early postoperative surveillance period. Nevertheless, all follow-up data used in this study were collected during in-person visits at our institution, ensuring consistency and data quality. These considerations support the assumption that censoring in this cohort was non-informative and unlikely to have introduced systematic survival bias. All in all, our results emphasize that, although the increased administration of systemic therapy has an undeniable role in the broader management of CCA, perioperative optimization, particularly the decline in FTR rates, has been the most influential driver of survival improvement in our surgically treated cohort. Ultimately, liver resection remains the primary treatment for resectable *de novo* pCCA, whereas liver transplantation could offer a survival advantage in carefully selected patients with unresectable disease who meet strict transplant criteria. For patients with borderline-resectable tumors, particularly those with vascular involvement but who ineligible for transplantation, surgical exploration remains justified, because outcomes are significantly better than in patients who are unable to proceed to curative therapy.[27]

This study has several limitations. First, the retrospective design introduces inherent limitations, including potential biases resulting from missing data, variability in documentation practices, and unmeasured confounding factors. In particular, a temporal bias must be acknowledged. Earlier records might underestimate complication rates and rescue events because of limited documentation. In addition, variation in the use of adjuvant therapy across eras introduces a potential bias in the interpretation of long-term oncological outcomes. Patients treated during earlier eras might not have received adjuvant chemotherapy despite meeting contemporary guidelines, reflecting a historical bias resulting from evolving treatment standards. Furthermore, the potential for immortal time bias warrants consideration, given the observed increase in the time interval from diagnosis to surgery in the most recent cohort. This likely reflects a more meticulous preoperative workup, including detailed radiological evaluation, multidisciplinary assessment, and functional liver testing. Moreover, the growing use of neoadjuvant therapy in this cohort inherently

prolongs the interval from diagnosis to resection, introducing potential for survivor bias, because only patients who tolerate and respond to therapy proceed to surgery. Importantly, however, such delays would be expected to bias survival estimates against the most recent cohort, rather than in its favor, thereby minimizing, rather than inflating, the risk of immortal time bias in this context.

Conclusion

In conclusion, our findings suggest that the temporal improvement in OS following curative-intent resection for pCCA is primarily attributable to a marked reduction in FTR rates, even in the context of increasingly complex surgical procedures and a rising incidence of major complications. Although advances in systemic therapy have undoubtedly contributed to the evolving treatment landscape, their impact in our cohort was less pronounced, particularly when early postoperative mortality was excluded. Ultimately, the ability to successfully manage rather than merely prevent life-threatening complications has emerged as the cornerstone of progress in this high-risk surgical population. Moving forward, continued advances in perioperative infrastructure, multidisciplinary expertise, and patient optimization will be essential to further improve long-term outcomes in pCCA.

Affiliations

[1]*Department of Surgery, Division of Hepatobiliary and Pancreatic Surgery, Mayo Clinic, Minnesota, Rochester, MN, USA;* [2]*William J. von Liebig Center for Transplantation and Clinical Regeneration, Mayo Clinic, Rochester, MN, USA;* [3]*Department of Internal Medicine, Division of Gastroenterology and Hepatology, Mayo Clinic, Rochester, MN, USA;* [4]*Department of Immunology, Mayo Clinic, Rochester, MN, USA;* [5]*Department of Biochemistry and Molecular Biology, Mayo Clinic, Rochester, MN, USA;* [6]*Center for Physiology and Pharmacology, Medical University of Vienna, Vienna, Austria*

Abbreviations

AJCC, American Joint Committee on Cancer; ALBI, albumin–bilirubin score; ALT, alanine aminotransferase; APRI, aspartate-aminotransferase-platelet-ratio-index; AST, aspartate aminotransferase; CA19-9, carbohydrate antigen 19-9; CCA, cholangiocarcinoma; CTx, chemotherapy; ECOG, Eastern Cooperative Oncology Group; ERCP, endoscopic retrograde cholangiopancreatography; FLR, future liver remnant; FTR, failure-to-rescue; HCC, hepatocellular carcinoma; ICU, Intensive care unit; INR, international normalized ratio; IR, interventional radiology; ISGLS, International Study Group of Liver Surgery; LN, lymph node; NA, not available; NR, not reached; OS, overall survival; pCCA, perihilar cholangiocarcinoma; PHFL, posthepatectomy liver failure; PSC, primary sclerosing cholangitis; PTCD, percutaneous transhepatic cholangiographic drainage; PVE, portal vein embolization; RFS, recurrence-free survival; T, tumor.

Financial support

The present study was a retrospective analysis that did not receive any financial support from external grants.

Conflicts of interest

The authors declare no conflicts of interest that pertain to this work.

Please refer to the accompanying ICMJE disclosure forms for further details.

Authors' contributions

Conceptualization, data curation, formal analysis: YD, ZL, VP, JEE, SGW, DMN, RLS, SII, GJG, PPS. Investigation and methodology: YD, ZL, SII, GJG, PPS. Writing – original draft: YD, PPS. Writing – review and editing: YD, ZL, VP, JEE, SGW, DMN, RLS, SII, GJG, PPS. Supervision: SII, GJG, PPS.

Acknowledgements

YD was a recipient of the IHPBA Kenneth Warren Fellowship while conducting this retrospective study.

Supplementary data

Supplementary data to this article can be found online at https://doi.org/10.1016/j.jhepr.2025.101615.

References

Author names in bold designate shared co-first authorship

[1] Sugiura T, Uesaka K, Okamura Y, et al. Major hepatectomy with combined vascular resection for perihilar cholangiocarcinoma. BJS Open 2021;5:zrab064.

[2] Ilyas SI, Affo S, Goyal L, et al. Cholangiocarcinoma - novel biological insights and therapeutic strategies. Nat Rev Clin Oncol 2023;20:470–486.

[3] Jain AJ, Lendoire M, Haddad A, et al. Improved outcomes following resection of perihilar cholangiocarcinoma: a 27-year experience. Ann Surg Oncol 2025;32:4352–4362.

[4] Benzing C, Schmelzle M, Atik CF, et al. Factors associated with failure to rescue after major hepatectomy for perihilar cholangiocarcinoma: a 15-year single-center experience. Surgery 2022;171:859–866.

[5] Ratti F, Marino R, Catena M, et al. The failure to rescue factor: aftermath analyses on 224 cases of perihilar cholangiocarcinoma. Updates Surg 2023;75:1919–1939.

[6] Fong ZV, Hallet J. Failure to rescue after resection of perihilar cholangiocarcinoma: can the pig fatten if weighed more often? Ann Surg Oncol 2025;32:2258–2259.

[7] Franken LC, Schreuder AM, Roos E, et al. Morbidity and mortality after major liver resection in patients with perihilar cholangiocarcinoma: a systematic review and meta-analysis. Surgery 2019;165:918–928.

[8] Taghizadeh H, Dong Y, Gruenberger T, et al. Perioperative and palliative systemic treatments for biliary tract cancer. Ther Adv Med Oncol 2024;16:17588359241230756.

[9] Primrose JN, Fox RP, Palmer DH, et al. Capecitabine compared with observation in resected biliary tract cancer (BILCAP): a randomised, controlled, multicentre, phase 3 study. Lancet Oncol 2019;20:663–673.

[10] Pecorelli N, Capretti G, Sandini M, et al. Impact of sarcopenic obesity on failure to rescue from major complications following pancreaticoduodenectomy for cancer: results from a multicenter study. Ann Surg Oncol 2018;25:308–317.

[11] Varley PR, Geller DA, Tsung A. Factors influencing failure to rescue after pancreaticoduodenectomy: a national surgical quality improvement project perspective. J Surg Res 2017;214:131–139.

[12] Gleeson EM, Pitt HA, Mackay TM, et al. Failure to rescue after pancreatoduodenectomy: a transatlantic analysis. Ann Surg 2021;274:459–466.

[13] Ardito F, Famularo S, Aldrighetti L, et al. The impact of hospital volume on failure to rescue after liver resection for hepatocellular carcinoma: analysis from the HE.RC.O.LE.S. Italian Registry. Ann Surg 2020;272:840–846.

[14] Olthof PB, Bouwense SAW, Bednarsch J, et al. Failure to rescue after resection of perihilar cholangiocarcinoma in an international multicenter cohort. Ann Surg Oncol 2025;32:1762–1768.

[15] Kawakatsu S, Ebata T, Watanabe N, et al. Mild prognostic impact of postoperative complications on long-term survival of perihilar cholangiocarcinoma. Ann Surg 2022;276:146–152.

[16] van Kooten RT, Bahadoer RR, Peeters KCMJ, et al. Preoperative risk factors for major postoperative complications after complex gastrointestinal cancer surgery: a systematic review. Eur J Surg Oncol 2021;47:3049–3058.

[17] Higuchi R, Yazawa T, Uemura S, et al. Surgical outcomes for perihilar cholangiocarcinoma with vascular invasion. J Gastrointest Surg 2019;23:1443–1453.

[18] van Keulen AM, Buettner S, Besselink MG, et al. Primary and secondary liver failure after major liver resection for perihilar cholangiocarcinoma. Surgery 2021;170:1024–1030.

[19] Sparrelid E, Olthof PB, Dasari BVM, et al. Current evidence on post-hepatectomy liver failure: comprehensive review. BJS Open 2022;6:zrac142.

[20] Santol J, Kim S, Gregory LA, et al. An APRI+ALBI based multivariable model as preoperative predictor for posthepatectomy liver failure. Ann Surg 2023;281:861–871.

[21] Kelley RK, Ueno M, Yoo C, et al. Pembrolizumab in combination with gemcitabine and cisplatin compared with gemcitabine and cisplatin alone for patients with advanced biliary tract cancer (KEYNOTE-966): a randomised, double-blind, placebo-controlled, phase 3 trial. Lancet 2023;401:1853–1865.
[22] Oh DY, He AR, Bouattour M, et al. Durvalumab or placebo plus gemcitabine and cisplatin in participants with advanced biliary tract cancer (TOPAZ-1): updated overall survival from a randomised phase 3 study. Lancet Gastroenterol Hepatol 2024;9:694–704.
[23] Rizzo A, Brandi G. BILCAP trial and adjuvant capecitabine in resectable biliary tract cancer: reflections on a standard of care. Expert Rev Gastroenterol Hepatol 2021;15:483–485.
[24] De Bellis M, Mastrosimini MG, Conci S, et al. The prognostic role of true radical resection in perihilar cholangiocarcinoma after improved evaluation of radial margin status. Cancers 2022;14:6126.
[25] Shinohara K, Ebata T, Shimoyama Y, et al. A study on radial margin status in resected perihilar cholangiocarcinoma. Ann Surg 2021;273:572–578.
[26] Datta J, Willobee BA, Ryon EL, et al. Contemporary reappraisal of intraoperative neck margin assessment during pancreaticoduodenectomy for pancreatic ductal adenocarcinoma: a review. JAMA Surg 2021;156:489–495.
[27] **Dong Y**, **Li Z**, Podrascanin V, et al. Liver resection with and without vascular resection versus transplantation for de novo perihilar cholangiocarcinoma. Hepatology 2025 Jul 4. Published online July 4. https://doi.org/10.1097/HEP.0000000000001449.

Keywords: Major complications; 90-day mortality; Post-hepatectomy liver failure; Adjuvant therapy; Preoperative optimization; Rescue strategies.
Received 6 July 2025; received in revised form 12 September 2025; accepted 25 September 2025; Available online 3 October 2025

Journal of Hepatology, Volume 7

Supplemental information

Failure-to-rescue as a determinant of overall survival following resection for perihilar cholangiocarcinoma

Yawen Dong, Zhihao Li, Vanja Podrascanin, John E. Eaton, Sumera I. Ilyas, Gregory J. Gores, Susanne G. Warner, David M. Nagorney, Rory L. Smoot, and Patrick P. Starlinger

Failure-to-rescue as a determinant of overall survival following resection for perihilar cholangiocarcinoma

Yawen Dong, Zhihao Li, Vanja Podrascanin, John E. Eaton, Sumera I. Ilyas, Gregory J. Gores, Susanne G. Warner, David M. Nagorney, Rory L. Smoot, Patrick P. Starlinger

Table of contents

Table S1……………………………………………………………………………………2

Table S2……………………………………………………………………………………3

Table S3……………………………………………………………………………………4

Fig. S1………………………………………………………………………………………7

Fig. S2………………………………………………………………………………………8

Table S4……………………………………………………………………………………9

Table S5……………………………………………………………………………………12

Fig. S3………………………………………………………………………………………13

Cause of Death in Failure to Rescue (FTR) Patients (N=15)	Total (N=15)	2000-2010 (N=7)	2011-2017 (N=5)	2017-2024 (N=3)
Primary posthepatectomy liver failure (PHLF)	2 (13.3%)	1 (14.3%)	0 (0.0%)	1 (33.3%)
Secondary PHLF, due to:<br>- Sepsis (n=3)<br>- Portal vein thrombosis (n=1)	4 (26.7%)	1 (14.3%)	2 (40.0%)	1 (33.3%)
Non-PHLF-related causes:<br>- Sepsis (n=4)<br>- Hemorrhage (n=2)<br>- Thromboembolic events (n=3)	9 (60.0%)	5 (71.4%)	3 (60.0%)	1 (33.3%)

Table S1. Summary of causes of death in patients with failure to rescue (FTR).

Rescue Interventions	2000-2010 (n=29)	2011-2017 (n=35)	2018-2024 (n=49)	Total (n=113)
ICU based rescue measures:	9 (31.0%)	11 (31.4%)	7 (14.3%)	27 (23.9%)
• Cardiorespiratory	3	4	3	10
• Bile leak/ PHLF	4	3	2	9
• Sepsis	1	1	1	3
• Bleeding	1	3	1	5
Relaparotomy	6 (20.7%)	11 (31.4%)	5 (10.2%)	22 (19.5%)
• Bile leak	3	4	3	10
• Intraabdominal hemorrhage	3	5	1	9
• Hepatic artery/ portal vein thrombosis	0	2	0	2
• Fascia dehiscence	0	0	1	1
Endoscopy based procedures:	0 (0.0%)	3 (8.6%)	2 (4.1%)	5 (4.4%)
• ERCP for bile leak	0	0	1	1
• EGD for GI bleeding	0	3	1	4
IR vascular procedures (e.g. coil embolization, PV stenting)	1 (3.5%)	1 (2.9%)	7 (14.3%)	9 (8.0%)
• Hepatic artery pseudoaneurysm	1	1	3	5
• Portal vein thrombosis	0	0	2	2
• Bleeding from right inferior phrenic artery	0	0	1	1
• Bleeding from gastroduodenal artery	0	0	1	1
IR PTCD placement for bile leak	3 (10.3%)	2 (5.7%)	15 (30.6%)	20 (17.7%)
Image guided drainage (e.g. CT/ US guided, paracentesis, thoracocentesis)	10 (34.5%)	7 (20.0%)	13 (26.5%)	30 (26.5%)
• Bilioma	2	1	4	7
• Abscess	5	5	7	17
• Pleural effusion	2	0	0	2
• Ascites	1	0	1	2
• Infected hematoma	0	1	1	2

Table S2. Rescue interventions following major complications in resected pCCA patients by era (2000-2010 vs 2011-2017 vs 2018-2024). Percentages are calculated per time period. PHLF = Post-hepatectomy liver failure, ERCP = Endoscopic retrograde cholangiopancreatography, EGD = Esophagogastroduodenoscopy, GI = Gastrointestinal, IR = Interventional radiology, PV = Portal vein.

Variable	Major Complications (N=81)	Rescued group (N=66)	Failure to rescue group (N=15)	p value
Sex				0.331
Male	52 (64.2%)	44 (66.7%)	8 (53.3%)	
Female	29 (35.8%)	22 (33.3%)	7 (46.7%)	
Age (years)	68.0 (56.5-73.0)	68.0 (57.0-73.0)	69.0 (57.0-75.5)	0.212
BMI (kg/m^2)	25.6 (23.2-30.0)	25.1 (23.0-28.7)	30.0 (25.8-39.4)	0.002
ECOG				0.250
• 0	58 (71.6%)	49 (74.2%)	9 (60.0%)	
• > 0	23 (28.4%)	17 (25.8%)	6 (40.0%)	
Bilirubin (mg/dl)	0.9 (0.5-2.6)	0.9 (0.5-2.6)	1.1 (0.6-2.6)	0.209
APRI+ALBI	-2.17 (-2.49 - -1.55)	-2.24 (-2.54 - -1.52)	-2.20 (-2.42 - -0.73)	0.023
Prothrombin time (sec)	11.5 (10.5-12.78)	11.6 (10.9-12.8)	11.3 (10.2-12.8)	0.804
Platelets (x10^9/L)	251.0 (200.0-322.0)	242.0 (197.0-312.0)	243.0 (180.5-379.0)	0.597
INR	1.0 (1.0-1.1)	1.1 (1.0-1.2)	1.0 (1.0-1.2)	0.902
AST (U/L)	54.0 (34.5-83.5)	59.0 (35.0-88.0)	57.0 (43.0-112.0)	0.316
ALT (U/L)	61.0 (37.8-104.5)	61.0 (37.0-110.0)	67.0 (48.0-105.0)	0.681
Albumin (g/dl)	4.0 (3.7-4.2)	4.0 (3.7-4.3)	3.9 (3.6-4.1)	0.038
CA19-9 (U/ml)	108.5 (36.0-372.8)	107.0 (38.0-374.0)	239.0 (36.0-713.0)	0.635
Bismuth-Corlette				0.063
• I	2 (2.5%)	1 (1.5%)	1 (6.7%)	
• II	5 (6.2%)	5 (7.6%)	0 (0.0%)	
• IIIa	40 (49.4%)	35 (53.0%)	5 (33.3%)	
• IIIb	27 (33.3%)	18 (27.3%)	9 (60.0%)	
• IV	7 (8.6%)	7 (10.6%)	0 (0.0%)	
PreOP stenting	70 (86.4%)	55 (83.3%)	15 (100%)	0.089
Portal vein embolization	16 (19.8%)	15 (22.7%)	1 (6.7%)	0.158
Time period of surgery				0.181
• 2000-2010	24 (29.6%)	17 (25.8%)	7 (46.7%)	
• 2011-2017	26 (32.1%)	21 (31.8%)	5 (33.3%)	
• 2018-2024	31 (38.3%)	28 (42.4%)	3 (20.0%)	

Type of liver resection				0.033
• Right hepatectomy	26 (32.1%)	22 (33.3%)	4 (26.7%)	
• Extended right hepatectomy	24 (29.6%)	21 (31.8%)	3 (20.0%)	
• Left hepatectomy	19 (23.5%)	11 (16.7%)	8 (53.3%)	
• Extended left hepatectomy	9 (11.1%)	9 (13.6%)	0 (0.0%)	
• Central hepatectomy	3 (3.7%)	3 (4.6%)	0 (0.0%)	
Vascular reconstruction	19 (23.5%)	16 (24.2%)	3 (20.0%)	0.726
TNM Stage (AJCC 8th edition)				0.671
• Stage 0	1 (1.2%)	1 (1.5%)	0 (0.0%)	
• Stage 1	10 (12.3%)	9 (13.6%)	1 (6.7%)	
• Stage 2	29 (35.8%)	25 (37.9%)	4 (26.7%)	
• Stage 3	35 (43.2%)	26 (39.4%)	9 (60.0%)	
• Stage 4	6 (7.4%)	5 (7.6%)	1 (6.7%)	
Tumor size (cm)	2.9 (2.3-4.0)	2.8 (2.2-3.4)	3.5 (2.5-5.0)	0.513
Lymph node (LN) status				0.454
• N0	39 (48.1%)	33 (50.0%)	6 (40.0%)	
• N1	36 (44.4%)	27 (40.9%)	9 (60.0%)	
• N2	2 (2.5%)	2 (3.0%)	0 (0.0%)	
• Nx	4 (4.9%)	4 (6.1%)	0 (0.0%)	
Histologic grade				0.868
• G1	12 (14.8%)	9 (13.6%)	0 (0.0%)	
• G2	30 (37.0%)	26 (39.4%)	3 (20.0%)	
• G3	33 (40.7%)	26 (39.4%)	7 (46.7%)	
• G4	5 (6.2%)	4 (6.1%)	1 (6.7%)	
• NA	1 (1.2%)	1 (1.5%)	0 (0.0%)	
Resection margin				0.489
• R0	65 (80.2%)	52 (78.8%)	13 (86.7%)	
• R1	16 (19.8%)	14 (21.2%)	2 (13.3%)	
Estimated blood loss (ml)	700.0 (500.0-1400.0)	700.0 (500.0-1500.0)	800.0 (350.0-1150.0)	0.981
PostOP stay (days)	12.0 (9.0-19.0)	12.0 (9.0-19.0)	7.0 (6.0-16.0)	0.214

Neoadjuvant therapy	9 (11.1%)	8 (12.1%)	1 (6.7%)	0.544
PHLF Grade B/ C	34 (42.0%)	27 (40.9%)	7 (46.7%)	0.530

Table S3. Comparison of major complications, 90-day mortality, and failure to rescue (FTR) rates between rescued and failure to rescue (FTR) patients following major hepatectomy for pCCA.

Continuous variables are presented as medians with interquartile ranges (IQRs) and were compared using the Mann-Whitney U test for pairwise comparisons. Categorical variables are reported as counts and percentages and were compared using the Chi-square test or Fisher's exact test, as appropriate. A p-value <0.05 was considered statistically significant; significant p-values are indicated in bold.

BMI body mass index, ECOG Eastern Cooperative Oncology Group, APRI Aspartate Aminotransferase to Platelet Ratio Index, ALBI Albumin-Bilirubin Score, INR international normalized ratio, AST Aspartate Aminotransferase, ALT Alanine Aminotransferase, CA7❺_❺Carbohydrate Antigen 19-9, PVE portal vein embolization, pCCA.perihilar cholangiocarcinoma.

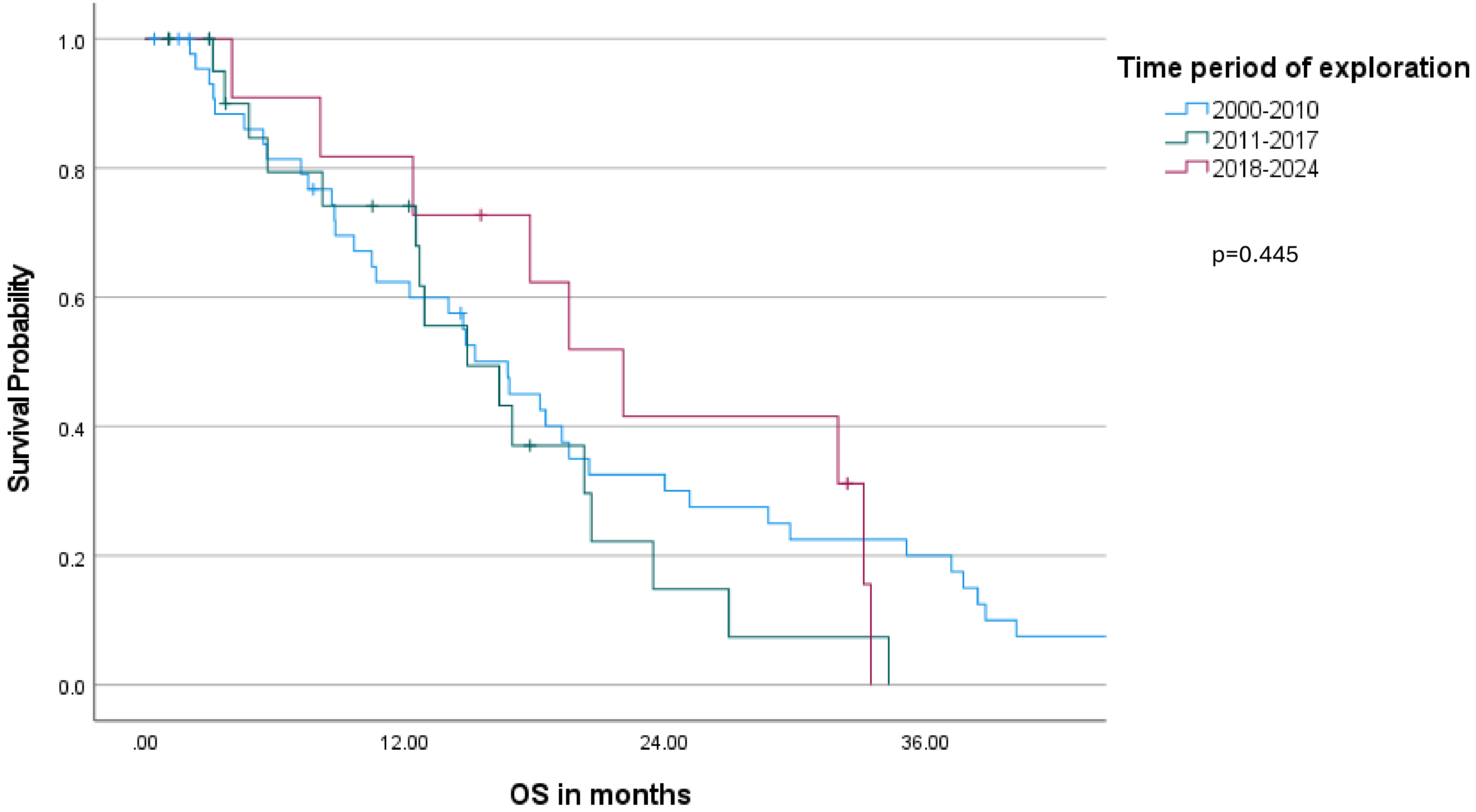

Number at risk

2000-2010	46	26	12	8
2011-2017	24	13	2	0
2018-2024	11	9	4	0

Fig. S1. Kaplan Meier Curve demonstrating OS of all pCCA dropout patients stratified by time period of surgical exploration.

Statistical analysis was performed using Kaplan-Meier survival estimates and log-rank test. A p-value <0.05 was considered statistically significant, and significant p-values are indicated in bold, while non-significant p-values are shown without formatting.

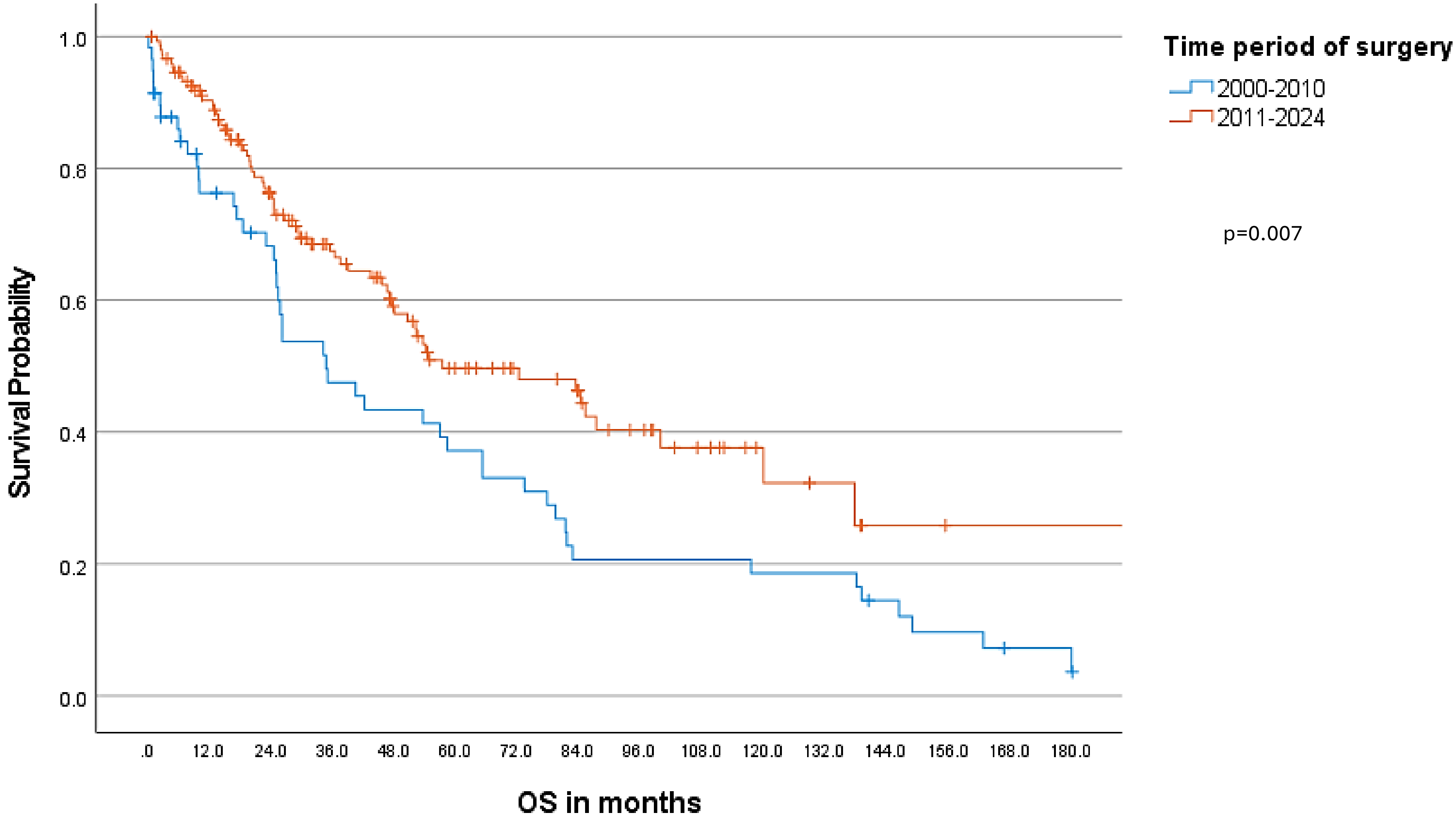

Number at risk

2000-2010	58	39	33	23	21	18	16	10	10	10	9	9	6	4	2	1
2011-2024	149	123	91	67	52	38	30	25	18	12	7	5	2	1	1	1

Fig. S2. Assessment of the impact of adjuvant therapy on OS in resected pCCA patients. Comparison between a historical cohort (2000–2010) and a recent cohort (2011–2024), using 2010 as the cutoff year to reflect the increasing adoption of adjuvant therapy thereafter. Statistical analysis was performed using Kaplan-Meier survival estimates and log-rank test. A p-value <0.05 was considered statistically significant, and significant p-values are indicated in bold, while non-significant p-values are shown without formatting.

Variable	No adjuvant therapy (N=111)	Adjuvant therapy (N=96)	p value
Sex			0.797
Male	69 (62.2%)	58 (60.4%)	
Female	42 (37.8%)	38 (39.6%)	
Age (years)	68.0 (60.0-73.5)	63.0 (52.0-71.0)	0.001
BMI (kg/m^2)	25.6 (22.6-28.9)	25.2 (22.8-29.7)	0.870
ECOG			0.148
0	76 (68.5%)	77 (80.2%)	
> 0	35 (31.5%)	19 (19.8%)	
Bilirubin (mg/dl)	1.1 (0.6-3.2)	0.9 (0.5-2.1)	0.132
APRI+ALBI	-1.85 (-2.42 - -1.35)	-2.23 (-2.62 - -1.65)	0.037
Prothrombin time (sec)	11.6 (10.8-12.7)	11.7 (11.2-12.2)	0.099
Platelets (x10^9/L)	243.0 (197.5-307.0)	256.0 (197.0-307.0)	0.919
INR	1.0 (1.0-1.2)	1.0 (1.0-1.1)	0.204
AST (U/L)	62.0 (45.0-87.5)	51.0 (34.0-84.0)	0.103
ALT (U/L)	69.0 (48.5-111.5)	65.0 (44.0-119.0)	0.794
Albumin (g/dl)	3.9 (3.6-4.2)	4.1 (3.8-4.3)	0.046
CA19-9 (U/ml)	145.0 (36.0-514.0)	73.0 (22.0-259.0)	0.181
Bismuth-Corlette			0.010
I	2 (1.8%)	3 (3.1%)	
II	3 (2.7%)	8 (8.3%)	
IIIa	57 (51.4%)	31 (32.3%)	
IIIb	45 (40.5%)	42 (43.8%)	
IV	4 (3.6%)	12 (12.5%)	
PreOP stenting	103 (92.8%)	83 (86.5%)	0.132
Portal vein embolization	13 (11.7%)	11 (11.5%)	0.955
Time period of surgery			<0.001
2000-2010	45 (40.6%)	13 (13.5%)	
2011-2017	42 (37.8%)	31 (32.3%)	
2018-2024	24 (21.6%)	52 (54.2%)	
Type of liver resection			0.068
Right hepatectomy	43 (38.7%)	22 (22.9%)	
Extended right hepatectomy	18 (16.3%)	20 (20.8%)	

Left hepatectomy<br>Extended left hepatectomy<br>Central hepatectomy	40 (36.0%)<br>8 (7.2%)<br>2 (1.8%)	36 (37.5%)<br>16 (16.7%)<br>2 (2.1%)	
Vascular reconstruction	17 (15.3%)	20 (20.8%)	0.301
TNM Stage (AJCC 8th edition)			0.020
Stage 0	1 (0.9%)	0 (0.0%)	
Stage 1	16 (14.4%)	10 (10.4%)	
Stage 2	42 (37.9%)	26 (27.0%)	
Stage 3	49 (44.1%)	54 (56.3%)	
Stage 4	3 (2.7%)	6 (6.3%)	
Tumor size (cm)	2.7 (2.2-3.5)	2.9 (2.3-3.6)	0.071
Lymph node (LN) status			<0.001
N0	71 (64.0%)	31 (32.3%)	
N1	39 (35.1%)	55 (57.3%)	
N2	1 (0.9%)	2 (2.1%)	
Nx	0 (0.0%)	8 (8.3%)	
Histologic grade			0.040
G1	11 (9.9%)	13 (13.6%)	
G2	46 (41.4%)	51 (53.1%)	
G3	48 (43.2%)	31 (32.3%)	
G4	5 (4.5%)	1 (1.0%)	
NA	1 (0.9%)	0 (0.0%)	
Resection margin			0.002
R0	98 (88.3%)	68 (70.8%)	
R1	13 (11.7%)	28 (29.2%)	
Estimated blood loss (ml)	700.0 (425.0-1225.0)	600.0 (350.0-1200.0)	0.921
PostOP stay (days)	8.0 (5.0-15.0)	6.0 (4.0-10.0)	0.004
Neoadjuvant therapy	12 (10.8%)	11 (11.5%)	0.882
PHLF Grade B/ C	28 (25.2%)	9 (9.4%)	0.003
Major complications	55 (49.5%)	26 (27.1%)	<0.001
90d mortality	15 (13.5%)	0 (0.0%)	<0.001

Table S4. Baseline characteristics and clinicopathological parameters of pCCA patients stratified by receipt of adjuvant therapy. Continuous variables are presented as medians with interquartile ranges (IQRs) and were compared using the Mann-Whitney U test for pairwise comparisons. Categorical variables are reported as counts and percentages and were compared using the Chi-square test or Fisher's exact test, as appropriate. A p-value <0.05 was considered statistically significant; significant p-values are indicated in bold.

Time period	No Adjuvant Therapy Cohort (N=111)				Adjuvant Therapy Cohort (N=96)			
	Low risk & major complications (N=25)	Low risk w/o major complications (N=30)	High risk & major complications (N=30)	High risk w/o major complications (N=26)	Low risk & major complications (N=5)	Low risk w/o major complications (N=17)	High risk & major complications (N=21)	High risk w/o major complications (N=53)
2000-2010	9 (36.0%)	9 (30.0%)	13 (43.4(%)	14 (58.9%)	0 (0.0%)	0 (0.0%)	2 (9.5%)	11(20.8%)
2011-2017	9 (36.0%)	16 (53.3%)	10 (33.3%)	7 (26.9%)	2 (40.0%)	6 (35.3%)	5 (23.8%)	18 (34.0%)
2018-2024	7 (28.0%)	5 (16.7%)	7 (23.3%)	5 (19.2%)	3 (60.0%)	11 (64.7%)	14 (66.7%)	24 (45.2%)

Table S5. Distribution of patients in the adjuvant therapy cohort (N=96) and no adjuvant therapy cohort (N = 111), stratified by oncological risk profile (low vs. high risk) and presence of major postoperative complications across surgical time periods. Risk categorization was based on established adverse pathological features (e.g., nodal positivity, R1 resection, high-grade tumors), and major complications were defined as Clavien-Dindo grade ≥ III.

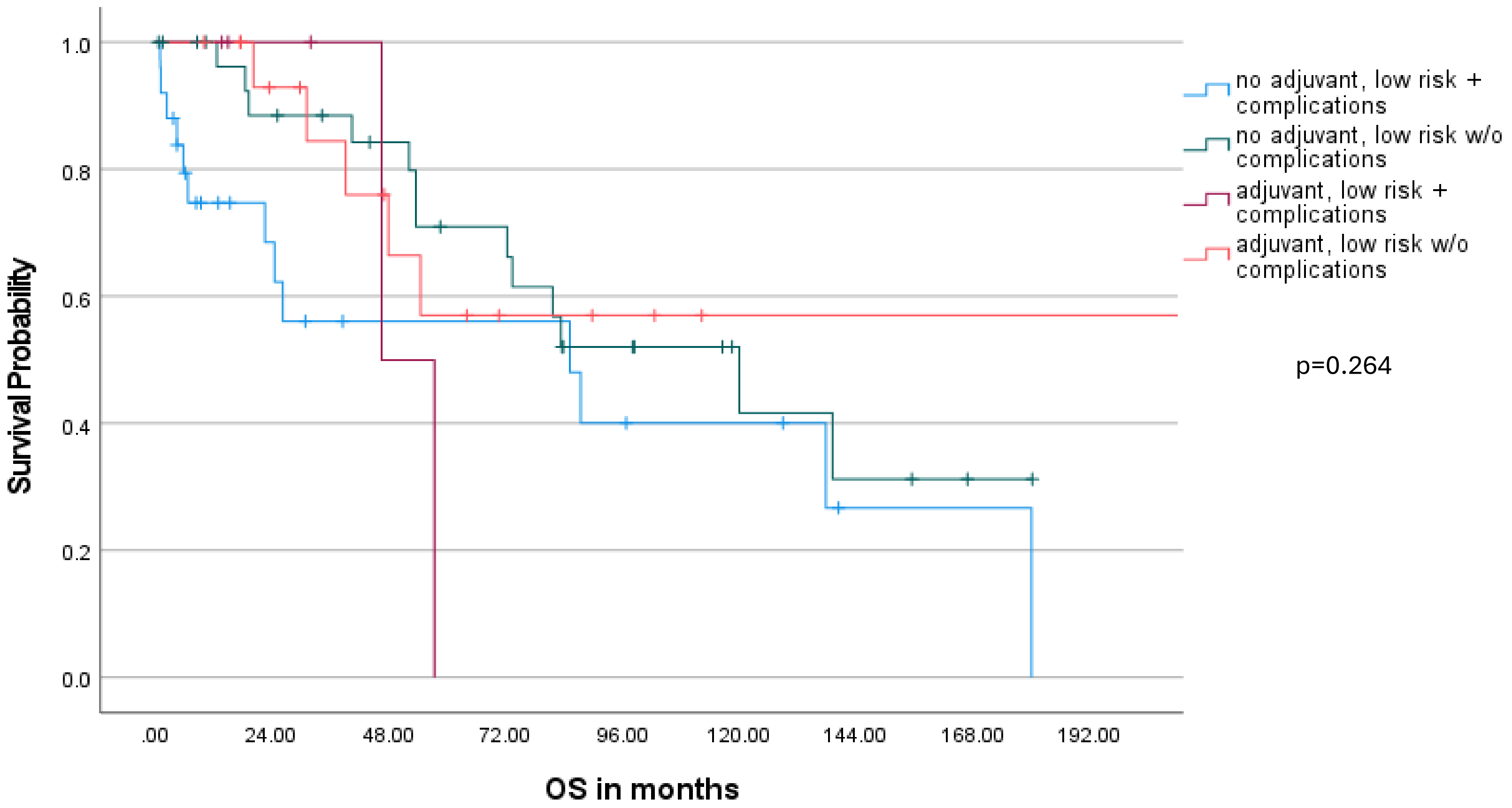

Number at risk

No adjuvant, low risk & complications	25	11	7	7	5	4	1	1	0
No adjuvant, low risk w/o complications	30	23	19	15	9	4	3	1	0
Adjuvant, low risk & complications	5	3	1	0	0	0	0	0	0
Adjuvant, low risk w/o complications	17	12	7	4	3	1	1	1	1

Fig. S3. Kaplan Meier Curve of OS in low risk profile patients stratified by receipt of adjuvant therapy and major complications. Statistical analysis was performed using Kaplan-Meier survival estimates and log-rank test. A p-value <0.05 was considered statistically significant, and significant p-values are indicated in bold, while non-significant p-values are shown without formatting.
